# Buyang Huanwu Decoction: A Traditional Chinese Medicine, Promotes Lactate-Induced Angiogenesis in Experimental Intracerebral Hemorrhage

**DOI:** 10.1155/2022/4063315

**Published:** 2022-10-30

**Authors:** Jing Zhou, Hao Guo, Ali Yang, Tao Liu, Pengfei Li, Hanjin Cui, Yang Wang, Tao Tang

**Affiliations:** ^1^Institute of Integrative Medicine, Department of Integrated Traditional Chinese and Western Medicine, Xiangya Hospital, Central South University, Changsha 410008, Hunan, China; ^2^Shanxi Province Academy of Traditional Chinese Medicine, Shanxi Province Hospital of Traditional Chinese Medicine, Taiyuan 030012, Shanxi, China; ^3^National Clinical Research Center for Geriatric Disorders, Xiangya Hospital, Central South University, Changsha 410008, Hunan, China; ^4^Department of Anesthesiology, Shanxi Provincial People's Hospital, Affiliate of Shanxi Medical University, Taiyuan 030000, Shanxi, China; ^5^Department of Neurology, Henan Province People's Hospital, Zhengzhou 450003, Henan, China; ^6^Department of Gerontology, Traditional Chinese Medicine Hospital Affliate to Xinjiang Medical University, Urumqi 830000, Xinjiang, China

## Abstract

Identifying the underlying mechanisms and exploring effective therapies for intracerebral hemorrhage (ICH) are urgently needed. Here, we aim to elucidate the potential roles and underlying mechanisms of Buyang Huanwu decoction (BYHWD) in ICH. In the first set of experiments, rats were randomly divided into five groups: Sham, ICH, ICH + sodium oxamate (OXA), ICH + BYHWD, and ICH + BYHWD + OXA. The lactate level around the hematoma was evaluated. PCNA^+^/vWF^+^ nuclei were observed. Additionally, an online bioinformatics analysis tool was used to predict the BYHWD druggable targets related to angiogenesis. Then, we validated these predictions. In the second set, exogenous sodium L-lactate (Lac) was infused into the intact brains of rats. Rats were randomly divided into three groups: Sham, Lac, and Lac + YC-1. The numbers of PCNA^+^/vWF^+^ nuclei and the expression of HIF-1*α* and VEGF were evaluated. In the first set of experiments, compared with the ICH group, the BYHWD group exhibited significantly increased numbers of PCNA^+^/vWF^+^ nuclei, and neurological dysfunction was markedly improved. Bioinformatics analysis revealed that the improvements caused by BYHWD indicated a role for the HIF-1*α* pathway. The HIF-1*α* and VEGF protein levels were upregulated after BYHWD administration. Moreover, we verified that lactate was involved in the predicted mechanisms. In the second set, lactate facilitated angiogenesis and HIF-1*α* and VEGF expression. Co-infusion with a HIF-1*α* inhibitor, YC-1, significantly inhibited these effects. Our data suggest that the pharmacological effects of BYHWD involve lactate-induced angiogenesis, these data may provide new evidence for its use in ICH.

## 1. Introduction

Intracerebral hemorrhage (ICH) is one of the most devastating forms of stroke and is characterized by high morbidity and mortality [1, 2]. Exploring effective therapies for ICH is urgently needed. However, until recently, advances in medical treatments have not been associated with strong improvements in outcomes [3–5]. Fortunately, the incorporation of herbal therapy into mainstream medical systems has been encouraged, and exploring therapeutic drugs from the library of herbal medicines offers a beacon of hope for ICH treatment [6, 7].

Buyang Huanwu decoction (BYHWD), a well-known traditional Chinese medicine (TCM), was first recorded in the ancient Chinese document “Yilin Gaicuo” by Qingren Wang in the Qing Dynasty. BYHWD consists of seven crude herbs: *Astragalus mongholicus* Bunge (Huang Qi), *Angellica sinensis* (Oliv) Diels (Dang Gui), *Prunus persica* (L.). Batsch (Tao Ren), *Carthamus tinctorius L*. (Hong Hua), *Paeonia lactiflora* Pall. (Chi Shao), *Ligusticum chuanxiong* Hort. (Chuan Xiong), and *Pheretima aspergillum* (E. Perrier) (Di Long). BYHWD has been demonstrated to be one of the most frequently prescribed formula for stroke treatment [8]. Accumulating evidence suggests that BYHWD can ameliorate neurological deficiencies in both experimental studies and clinical trials [9, 10]. Angiogenesis is a crucial self-repair process in the injured brain and involves the formation of new blood vessels from preexisting blood vessels [11, 12]. Enhancement of angiogenesis may be a promising therapeutic target for ICH. In our previous study, BYHWD has shown a valid protective response on ICH through its proangiogenic eﬀects, which were achieved by enhancing VEGFR2 phosphorylation through the PI3K/Akt signaling pathway [13]. Nevertheless, the multifaceted underlying molecular mechanisms by which BYHWD acts on ICH still need to be further elucidated.

Lactate has been reported to exert proangiogenic effects. In our previous study, we demonstrated that lactate accumulates around the hematoma and promotes angiogenesis in the brain after hemorrhagic injury [14]. Here, we were interested in studying the effects of BYHWD on lactate-induced angiogenesis. In the present study, BATMAN-TCM, an online bioinformatics analysis tool, was used to predict the signaling pathway related to BYHWD-induced angiogenesis. Furthermore, we verified that lactate was involved in the predicted mechanisms.

## 2. Materials and Methods

### 2.1. Animal Preparation and ICH Model Establishment

Male Sprague-Dawley (SD) rats (220∼250 g) were supplied by the Laboratory Animal Research Center of Central South University (CSU). The experimental protocol was established, performed in accordance with the guidelines, and approved by the Institutional Animal Care and Use Committee of CSU. The animals were housed in a controlled environment with a 12-h light/dark cycle. The rats were given free access to standard food and fresh water. The rats were anesthetized with pentobarbital sodium (50 mg/kg, i.p.). The animals were positioned on a stereotaxic frame (Stoelting Co.) and injected with 0.5 U collagenase (type VII, in 2.5 *μ*L of 0.9% sterile saline) into the right globus pallidus (coordinates: 1.4 mm posterior, 3.2 mm lateral to the bregma and 5.6 mm ventral to the cortical surface). After injection, the skin incision was closed with sutures. For the Sham group, 2.5 *μ*L of 0.9% sterile saline without collagenase was injected into the same site in the same manner.

### 2.2. BYHWD Preparation

All the dried herbs were purchased from and identified by the Chinese Medicinal Pharmacy of Xiangya Hospital (Changsha, China) (Table 1). The decoction was prepared and subjected to quality control, as previously described [15]. The blended supernatants were processed according to established standard procedures, and finally, the powder was dissolved in distilled water at a previously described concentration for intragastric administration [13, 15].

### 2.3. Qualitative Analysis of BYHWD

**Figure 1:**
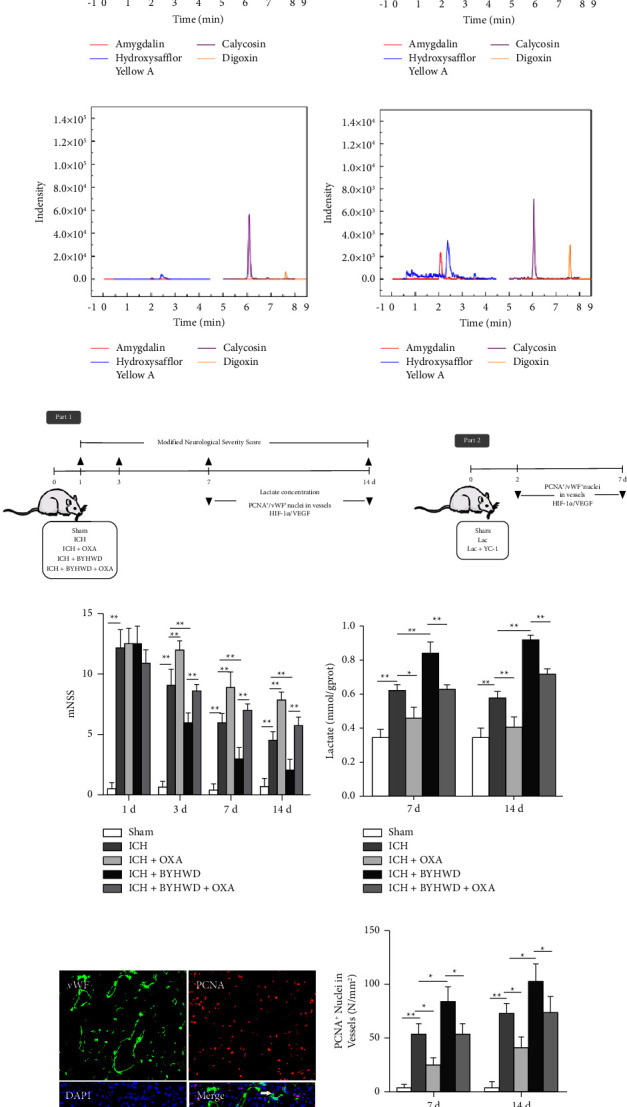
LC-MS analysis of three main components (amygdalin, hydroxysafflor yellow A and calycosin) of (a) standard agents, (b) decoction of BYHWD, (c) plasma sample of the ICH + BYHWD group and (d) brain sample of the ICH + BYHWD group. (e) The diagram of the experimental timeline. (f and g) The rats in the OXA group showed significantly higher mNSS (3 d, 7 d, and 14 d), and lower lactate levels (7 d and 14 d) than those in the ICH group. The neurological function of the BYHWD-treated rats markedly outperformed that of rats subjected to ICH on days 3, 7, and 14. BYHWD induced lactate accumulation in ICH. The rats in the BYHWD group showed better performances in the behavior test (3 d, 7 d and 14 d) and higher lactate levels than their inhibitor-treated counterparts, F: *n* = 6/group, G: *n* = 5/group. (h) Representative figures for PCNA^+^ nuclei located in vWF^+^ microvessels. (i) OXA significantly reduced the number of PCNA^+^/vWF^+^ cells at each time point after ICH. BYHWD significantly enhanced angiogenesis after ICH. Compared with those in the BYHWD-treated group, significantly decreased numbers of PCNA^+^ nuclei in vWF^+^ vessels were observed in the BYHWD + OXA-treated group, *n* = 5/group. ^*∗*^*p* < 0.05 and ^*∗∗*^*p* < 0.01. Scale bar = 100 *μ*m.

Qualitative analysis was carried out using LC-MS system (Shimadzu 8050). The standard reference materials were prepared: amygdalin, hydroxysafflor yellow A, calycosin, and digoxin (Yuanye Biotechnology Co., Ltd). For plasma samples, digoxin was added as an internal standard. 20 *μ*L of formic acid was added to 200 *μ*L of plasma samples. The samples were vortex-mixed for 10 s, and then vortex-mixed for 1 min and centrifuged for 15 min (13,000 r/min, 4°C) after being added with 800 *μ*L of acetonitrile. The obtained supernatants were dried in a nitrogen dryer, and diluted with 10% acetonitrile water for 200 *μ*L. They were vortex-mixed for 1 min, ultrasound for 2 min, and then centrifuged for 15 min (13,000 r/min, 4°C). The obtained supernatants were injected into the LC-MS for analysis. Brain tissue samples were homogenized, and 100 *μ*L of formic acid was added to precipitate the protein. The acetonitrile solution was added at 1 : 4 volume for extraction, and then vortex-mixed for 1 min and centrifuged for 10 min (4,000 r/min, 4°C). The supernatant was filtrated with a 0.22 *μ*m membrane and centrifuged for 15 min (13,000 r/min, 4°C). The obtained supernatants were dried in a nitrogen dryer, diluted with 10% acetonitrile water, and repeated the extraction above. The brain samples were also added to digoxin as the internal standard (Figures 1(a)–1(d)).

### 2.4. Experimental Design and Treatment Groups

All the rats were randomly assigned to the following experiments as described (Figure 1(e)).

The first set of experiments-To determine the effects of BYHWD in ICH and simultaneously identify the underlying mechanisms, rats were randomly divided into five groups: (1) Sham group: the rats received only vehicles; (2) ICH group: the rats were subjected to ICH and received vehicles in the corresponding site; (3) ICH + OXA group: the rats were subjected to ICH, received the lactate dehydrogenase inhibitor OXA (sodium oxamate, Sigma-Aldrich, aCSF, 50 mM, i.c.v., ALZET® Osmotic Pumps), and were intragastrically administered distilled water alone; (4) ICH + BYHWD group: the rats were subjected to ICH, given an intragastric administration of BYHWD, and received aCSF in the corresponding set; and (5) ICH + BYHWD + OXA group: the rats were subjected to ICH and received BYHWD and OXA. All the rats were euthanized on day 7 or 14 after ICH.

The second set of experiments - To verify the role of lactate in angiogenesis in the rat brain, rats were randomly assigned into the following groups: (1) Sham group: the rats received only vehicles; (2) Lac group: intact rats received an infusion of L-lactate (sodium L-lactate, Sigma-Aldrich, 0.9% sterile saline, 10 mM, ALZET® Osmotic Pumps) into the right globus pallidus; and (3) Lac + YC-1: some rats in the lactate group received YC-1 (Sigma-Aldrich, 1 mg/kg/d, intravenous injection) to inhibit HIF-1*α*. The rats were sacrificed on days 2 and 7 after the L-lactate infusion.

### 2.5. Sample Preparation

After the treatment period, rats were anesthetized with pentobarbital sodium. For morphological analysis, rats were transcardially perfused with 0.9% saline followed by an ice-cold 4% paraformaldehyde. The removed brains were then postfixed in 4% paraformaldehyde before dehydration and embedding with paraffin. For Western blot and lactate concentration analyses, rats had only been perfused with 0.9% saline, and tissues adjacent to the hematoma were immediately collected and stored in liquid nitrogen. Then the tissues were stored at −80°C until use.

### 2.6. Neurobehavioral Test

The neurobehavioral test was previously described [16, 17], and was evaluated by investigators who were blinded to the experimental groups. The modified neurological severity score (mNSS), containing motor, reflex, and sensorimotor integration tests, was performed on days 1, 3, 7, and 14 after ICH.

### 2.7. Measurement of Lactate Concentration

The concentration of lactate in the perihematomal regions was determined using a lactate assay kit with spectrophotometric and enzymatic methods (Nanjing Jiancheng Bioengineering Institute, China). The tissues were homogenized in 0.9% sterile saline in an ice bath (weight: volume = 1 : 9). The protein concentrations were measured using the bicinchoninic acid (BCA, Thermo Fisher) method. The lactate concentration is expressed as mmol/gprot.

### 2.8. Bioinformatics Analysis by BATMAN-TCM Combined with KEGG

To obtain the anchored related pathways associated with BYHWD, BATMAN-TCM (https://bionet.ncpsb.org/batman-tcm) combined with KEGG enrichment analysis was applied. The pinyin name of the formula (BU YANG HUAN WU TANG) was submitted to BATMAN-TCM for bioinformatics analysis.

### 2.9. Immunohistochemical Analysis

For the immunohistochemical analysis, sections were incubated with mouse anti-VEGF (1 : 200, Abcam) or mouse anti-HIF-1*α* (1 : 200, Novus), and then with biotinylated anti-mouse IgG (1 : 800, Santa Cruz) for 1 h at 37°C. The samples were then incubated with an avidin-biotin-peroxidase complex (1 : 100, Vector Laboratories) for 1 h at 37°C. Immunoreactivity was visualized with DAB.

To detect proliferating cerebral microvascular endothelial cells, the sections were incubated with mouse anti-PCNA (1 : 1400, Cell Signaling Technology) and rabbit anti-vWF (1 : 400, Dakon) for 1 h at 37°C. The following secondary antibodies were then used: fluorescein 488-conjugated anti-rabbit antibody (1 : 1000, Jackson ImmunoResearch) and Cy3-conjugated anti-mouse antibody (1 : 1000, Jackson ImmunoResearch). After washing with PBS, the sections were stained with DAPI for 2 min to reveal the location of the nucleus. PCNA^+^/vWF^+^ nuclei close to the hematoma were counted. The data were presented as the number of nuclei per mm^2^ (N/mm^2^). To determine whether VEGF and HIF-1*α* were expressed in the endothelial cells , tissue sections were incubated with mouse anti-VEGF (1 : 200, Abcam) or mouse anti-HIF-1a (1 : 100, Novus) with rabbit anti-vWF (1 : 400, Dakon).

### 2.10. Western Blot Analysis

For Western blot analysis, proteins were electrophoresed and transferred onto PVDF membranes (Millipore). The membranes were then blocked in 5% BSA for 2 h, and then incubated with primary antibodies as follows: mouse anti-VEGF (1 : 400, Abcam), mouse anti-HIF-1*α* (1 : 300, Novus), or mouse anti-*β*-actin (1 : 4000, SAB) with gentle shaking at 4°C overnight. A HRP-conjugated antimouse IgG (1 : 5000, Proteintech) was added and incubated for 2 h at room temperature. The specific reactions were visualized via the use of an enhanced chemiluminescent substrate (Thermo Fisher). The level of protein expression is presented relative to the levels of *β*-actin expression.

### 2.11. Statistical Analysis

The data are expressed as the mean ± SD. Data were analyzed by *t*-test or one-way ANOVA. Differences were considered significant at *p* < 0.05.

## 3. Results

### 3.1. BYHWD Treatment Improved Neurobehavioral Outcomes

Neurological function was evaluated by the mNSS (Figure 1(f)). The sham-operated animals were almost free of neurological impairments. On day 1, all the rats subjected to ICH showed similar neurological deficits. The rats in the OXA group (3 d, 7 d, and 14 d) showed a significantly higher mNSS than those in the ICH group. The neurological function of the BYHWD-treated rats markedly outperformed that of the ICH rats on days 3, 7, and 14. The rats in the BYHWD group (3 d, 7 d, and 14 d) showed better performances in the behavior test than their inhibitor-treated counterparts.

### 3.2. BYHWD Promoted Lactate Accumulation and Angiogenesis in ICH

The lactate content of the perihematomal tissues was significantly increased at days 7 and 14 after ICH (Figure 1(g)). OXA markedly suppressed the lactate levels. The BYHWD induced more lactate accumulation in the brains of the rats subjected to ICH, while the lactate levels in the brains of the rats treated with BYHWD + OXA were notably lower than those in the brains of the rats treated with BYHWD (Figure 1(g)).

The effects of BYHWD on angiogenesis in the brains of the rats after ICH were examined using double-labeling immunofluorescence (Figures 1(h) and 1(i)). PCNA^+^ nuclei in vWF^+^ vessels were rarely observed in either hemisphere of the sham-operated animals. In contrast, the number of newborn nuclei in vWF^+^ microvessels surrounding the hematoma following ICH increased on days 7 and 14, and OXA dramatically reduced the number of positive cells. BYHWD treatment significantly increased the number of newborn vessels (Figure 1(i)). To verify whether BYHWD promoted lactate-induced angiogenesis in ICH, OXA and BYHWD were administered to animals subjected to ICH. Compared with those of the BYHWD-treated group, significantly diminished PCNA^+^ nuclei in vWF^+^ vessels were observed surrounding the hematoma of the BYHWD + OXA-treated group (Figure 1(i)).

### 3.3. BATMAN-TCM for Pathway Enrichment Analysis

Enrichment analyses were implemented to predict the pathways associated with BYHWD that are related to angiogenesis after the ICH. According to KEGG enrichment analysis, the HIF-1*α* signaling pathway (adjusted *p*-value = 0.0308), one of the main angiogenesis-related pathways, was successfully enriched (Supplementary Table available here).

### 3.4. BYHWD Promoted Lactate-Induced Angiogenesis in ICH through the HIF-1*α*/VEGF Signaling Pathway

**Figure 2:**
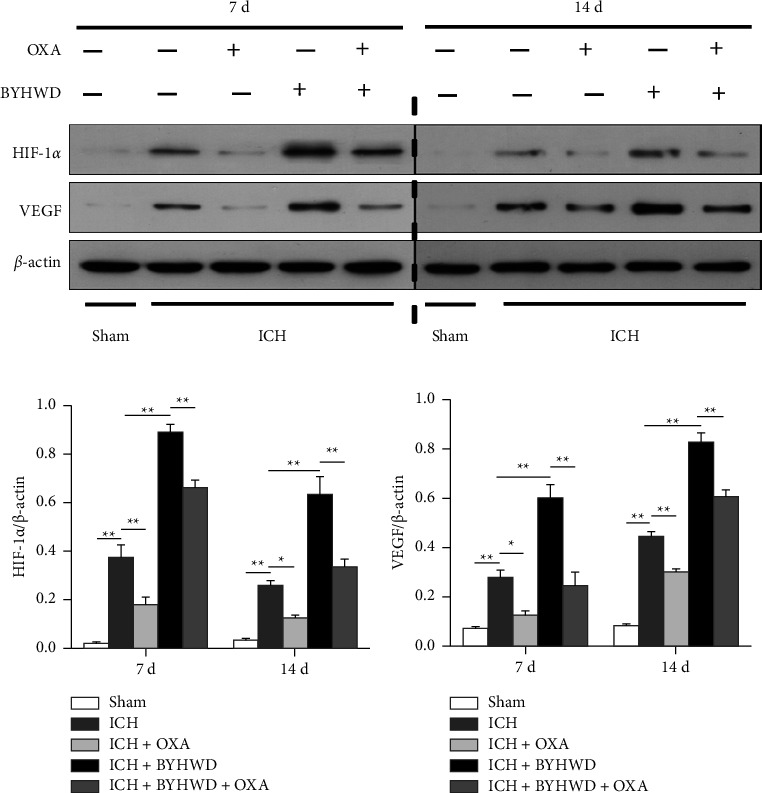
(a) Representative Western blot analysis of HIF-1*α* and VEGF expression in each group. (b and c) Following ICH, the expression of HIF-1*α* started to increase beginning on day 7, and then showed a moderate decrease at day 14; the levels of VEGF exhibited an increasing trend through days 7 to 14. OXA markedly decreased the expression of HIF-1*α* and VEGF at each time point. Compared with the ICH group, the BYWHD group exhibited significantly increased HIF-1*α* and VEGF levels, and OXA blocked these effects, *n* = 3/group. ^*∗*^*p* < 0.05 and ^*∗∗*^*p* < 0.01.

**Figure 3:**
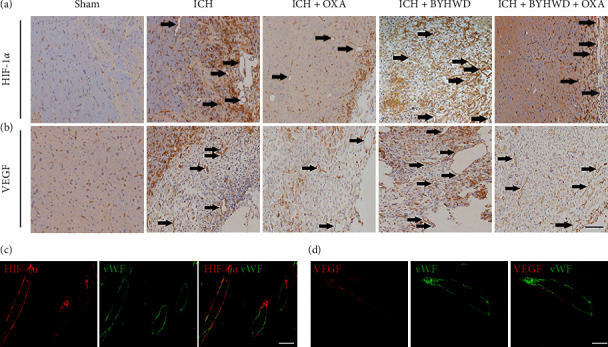
Representative images of (a) HIF-1*α* and (b) VEGF immunoreactivities on day 7 were shown. Barely any HIF-1*α* (a) and VEGF (b) immunoreactivity was found in the Sham group, and many HIF-1*α*^+^ (a) and VEGF^+^ (b) microvessels were detected around the hematoma after ICH in the enlarged profile. Immunofluorescence double labeling confirmed that (c) HIF-1*α* and (d) VEGF were localized to vWF-positive vessels. Scale bar = 100 *μ*m (a, b). Scale bar = 50 *μ*m (c, d).

To verify the bioinformatics prediction by BATMAN-TCM, Western blot and morphological analyses were executed. The Western blot analysis showed that the level of HIF-1*α* started to increase beginning on day 7 and then showed a moderate decline on day 14 after ICH (Figures 2(a) and 2(b)). The protein expression of VEGF showed an increasing trend from days 7 to 14 after ICH (Figures 2(a) and 2(c)). OXA markedly inhibited the protein expression of HIF-1*α* and VEGF at each time point. Compared with those in the ICH group, significantly increased HIF-1*α* and VEGF protein levels were found in the BYWHD group. To verify whether BYHWD promotes lactate accumulation, thereby inducing angiogenesis through the HIF-1*α*/VEGF signaling pathway, OXA was administered to ICH animals together with BYHWD. We found dramatically reduced protein levels of HIF-1*α* and VEGF in the BYHWD + OXA-treated group compared with the BYHWD-treated group (Figures 2(a)–2(c)). Immunohistochemical analysis showed that little HIF-1*α* or VEGF immunoreactivity was found in the Sham group (Figures 3 and 3, representative images were shown on day 7). Many HIF-1*α*^+^ and VEGF^+^ microvessels were detected surrounding the hematoma following ICH in the enlarged profile. Immunofluorescence double labeling confirmed that both HIF-1*α* and VEGF were localized to vWF-positive vessels (Figures 3 and 3(d)).

**Figure 4:**
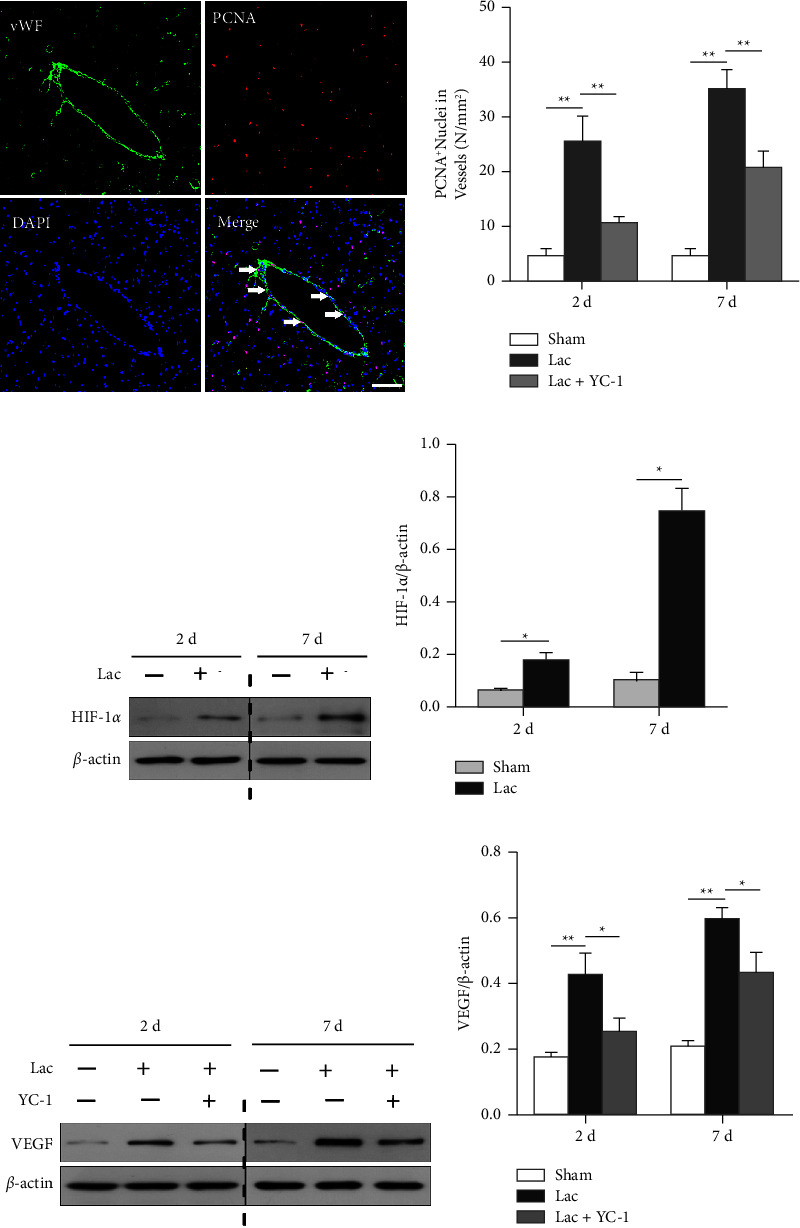
(a and b) L-lactate significantly increased the number of PCNA^+^/vWF^+^ nuclei. YC-1 blocked these effects, *n* = 5/group. (c) Representative figures for Western blot analysis of HIF-1*α*. (d) The expression levels of HIF-1*α* were strikingly increased after L-lactate infusion (2 d and 7 d), n = 3/group. (e) Representative figures of Western blot analysis of VEGF. (f) The expression levels of VEGF were strikingly increased after L-lactate infusion (2 d and 7 d), YC-1 blocked these effects, *n* = 3/group. ^*∗*^*p* < 0.05 and ^*∗∗*^*p* < 0.01. Scale bar = 100 *μ*m.

As described above, OXA blocked angiogenesis and HIF-1*α*/VEGF signaling pathway activation after ICH. The results suggested that the lactate/HIF-1*α*/VEGF signaling pathway played a vital role in BYHWD-mediated angiogenesis. To test this hypothesis, we infused L-lactate into intact rat brains. Significantly increased HIF-1*α* and VEGF protein levels were observed on days 2 and 7 after L-lactate infusion (Figures 4(c)–4(f)). To further confirm our hypothesis, we injected YC-1, an inhibitor of HIF-1*α*, into some L-lactate-treated rats. Western blot analysis showed that the levels of VEGF were notably decreased after YC-1 intervention (Figures 4(e) and 4(f)). Moreover, in the YC-1-treated brains, the lactate-induced increase in the number of PCNA^+^ nuclei in vWF^+^ vessels was significantly attenuated (Figures 4(a) and 4(b)).

## 4. Discussion

**Figure 5:**
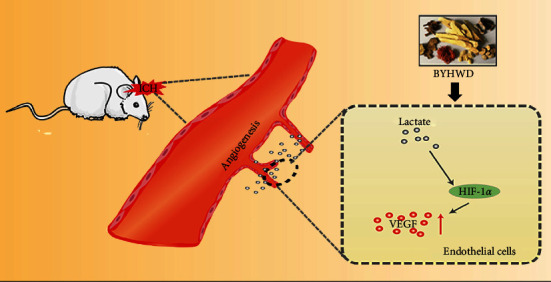
Buyang Huanwu decoction promotes lactate-induced angiogenesis in experimental ICH.

The major findings of the current study are as follows (Figure 5): (1) BYHWD promoted lactate accumulation and angiogenesis near the hematoma after ICH, (2) the proangiogenic effect of BYHWD was attenuated by reducing lactate accumulation in brains subjected to ICH, and (3) the HIF-1*α*/VEGF signaling pathway, which was predicted to be associated with of BYHWD, was proven to be involved in lactate-induced angiogenesis.

### 4.1. BYHWD Promotes Lactate Accumulation and Angiogenesis around the Hematoma after ICH

Lactate dehydrogenase (LDH), an important regulator of glycolysis, reversibly catalyzes the conversion of pyruvate to lactate. OXA is an established inhibitor of LDH via competition with pyruvate for its binding site on the enzyme [18]. The newborn nuclei in the vessels were significantly decreased by OXA after ICH (Figure 1(i)). This phenomenon suggested that lactate is crucial in the angiogenesis process during ICH. BYHWD promoted lactate accumulation and angiogenesis in the perihematomal regions of ICH, which suggested that the proangiogenic role of BYHWD might be related to its regulatory effect on lactate. Moreover, the proangiogenic effects of BYHWD were reversed by the coaddition of OXA (Figure 1(i)), which further confirms the conclusion that lactate plays a bridging role in BYHWD-mediated angiogenesis in ICH.

Lactate, which is produced during metabolic processes, is released from or enters into cells through monocarboxylate transporters (MCTs) [19]. Unlike ischemic stroke, ischemia and hypoxia are temporary and mild effects that occur adjacent to the hematoma after ICH [20]. Based on this evidence, on the 7^th^ and 14^th^ days, the accumulated lactate level surrounding the hematoma following ICH is probably not merely due to the lack of blood supply and anaerobic metabolism. Various cells, including DCX-positive new neurons, newborn nuclei in vWF^+^ vessels, and GFAP^+^ astrocytes, quickly proliferate from day 7 until day 14 after ICH [21–23]. Evidence has indicated that enhanced aerobic glycolysis occurs in cells undergoing rapid proliferation [24]. Thus, we speculated that the transformation may not necessarily be due to mitochondrial dysfunction, but the metabolic substances from aerobic glycolysis or oxidative metabolism may participate in different processes of cellular production and utilization.

The PI3K/Akt signaling pathway is a widely expressed system that is involved in many cellular processes, including inflammation and angiogenesis [25]. In addition to promoting HIF-1*α* expression, BYHWD has been confirmed to promote the recovery of ICH animals by enhancing VEGFR-2 phosphorylation through the PI3K/Akt signaling pathway [13]. Additionally, the major active constituent of Huang Qi (the sovereign drug of BYHWD), astragaloside IV, has been demonstrated to regulate the PI3K/Akt signaling pathway [26]. This evidence shows that the PI3K/Akt/HIF-1*α* signaling pathway is closely linked to BYHWD-mediated responses. In addition, the PI3K/Akt signaling pathway plays an important role in the tightly controlled regulation of metabolic adaptation that supports cell growth [27]. Akt causes a dose-dependent stimulation of lactate production in tumor cells. HIF-1a is expressed under the control of the PI3K/Akt pathway. The activation of Akt and HIF-1 resulting from PI3K activation promotes cell growth and survival by stimulating aerobic glycolysis [24, 27]. The PI3K/Akt/HIF-1a signaling pathway plays an essential role in changes in metabolic processes. In this study, we also showed that lactate accumulation in ICH can promote angiogenesis and HIF-1*α* expression and that this process can be promoted by BYHWD. Based on these findings, we speculated that the regulation of lactate accumulation by BYHWD in ICH may be due to the potential activation of the PI3K/Akt/HIF-1*α* signaling pathway.

### 4.2. Bioinformatics Instructional Approach to Predict the Therapeutic Mechanism of BYHWD

As the most frequently used TCM formula for stroke treatment, accumulating evidence suggests the brain protection role of BYHWD. Except for the whole recipe, there are several existing reports about the brain protection roles of the contained 7 herbs and their main ingredients. For instance, the Huangqi-Honghua combination and its main components, astragaloside IV and hydroxysafflor yellow A, could ameliorate cerebral infarction by antioxidant action in rats [28]. Astragaloside IV may alleviate early brain injury through antioxidative and antiapoptotic effects following experimental subarachnoid hemorrhage [29]. Moreover, astragaloside IV has the ability to maintain the integrity of the blood-brain barrier after ischemia or reperfusion [30]. Z-ligustilide (the main component of Dang Gui), could protect ischemia/reperfusion-induced brain injury by minimizing oxidative stress and antiapoptosis [31]. Furthermore, tetramethylpyrazine can ameliorate early brain injury after subarachnoid hemorrhage by suppressing neuronal apoptosis mediated by the PERK/Akt pathway [32]. The “multicompound, multitarget”-based BYHWD shows marked advantages in brain protection. However, the sophisticated improvement mechanisms of the multitarget-based TCM make it challenging to elucidate the proangiogenic roles of BYHWD. Fortunately, with advances in computational methods, systematic bioinformatics methods have been proposed to predict the targets of TCM. With the help of BATMAN-TCM [33, 34], a comprehensive and systematic online database for pharmacology analyses, the main angiogenesis-associated therapeutic target, the HIF-1*α* pathway, was anchored by KEGG enrichment analysis.

HIF-1*α* is the inducible subunit of HIF-1 and is the initial point and hub mediator of angiogenesis. Once activated, HIF-1*α* translocates into the nucleus and binds to the hypoxia response elements of several target genes. As one of the HIF-1*α* target genes, VEGF is the most potent angiogenic factor [35, 36]. The activation further facilitates the proliferation and migration of vascular endothelial cells during the process of angiogenesis. In this study, bioinformatics prediction and the subsequent morphology and Western blot analyses showed that BYHWD promotes angiogenesis by activating the HIF-1*α*/VEGF signaling pathway in ICH. The protein expression of HIF-1*α* and VEGF was attenuated by OXA in ICH brains, which suggested that BYHWD-induced lactate accumulation is involved in the activation process. A previous study also reported that lactate can activate HIF-1*α* in normoxic endothelial cells and result in the increased expression of angiogenesis-associated VEGFR2 [37]. In the second set of experiments, both exogenous lactate and the HIF-1*α* inhibitor-YC-1, were used. The numbers of PCNA^+^/vWF^+^ newborn nuclei and the protein levels of VEGF were increased after lactate perfusion, but these responses were blocked by YC-1. The results further verify the bioinformatics results that the HIF-1*α*/VEGF signaling pathway is essential in BYHWD-mediated angiogenesis after ICH and that lactate is involved in this process.

## 5. Conclusion

This study has shown that BYHWD could promote lactate accumulation, which might induce angiogenesis by activating the HIF-1*α*/VEGF signaling pathway after ICH. Moreover, the method of bioinformatics prediction together with molecular biological validation in the present study may provide a novel research strategy for the mechanism exploration of TCM.

## Figures and Tables

**Table 1:** Components of the Buyang Huanwu decoction.

Scientific name	Chinese name	Medicinal part	Place of production	Batch number
*Astragalus mongholicus* Bunge	Huang Qi	Root	Nei meng	168110807
*Angellica sinensis* (Oliv) Diels.	Dang Gui	Root	Gan su	16111801
*Paeonia lactiflora* Pall.	Chi Shao	Root	Si chuan	16101908
*Prunus persica* (L.) Batsch	Tao Ren	Seed	Si chuan	16080810
*Carthamus tinctorius L*.	Hong Hua	Flower	Xin jiang	16121207
*Ligusticum chuanxiong* Hort.	Chuan Xiong	Root	Si chuan	16120102
*Pheretima aspergillum* (E. Perrier)	Di Long	Whole animal	Guang dong	16090702

## Data Availability

The data that support the findings of this study are available from the corresponding author upon request.

## Supplementary Materials

Supplementary Materials KEGG pathway enrichment analysis result of BYHWD by using BATMAN-TCM.
