# Supplementary material for: Buyang Huanwu Decoction: A Traditional Chinese Medicine, Promotes Lactate-Induced Angiogenesis in Experimental Intracerebral Hemorrhage
Source: Evid Based Complement Alternat Med. 2022 Oct 30;2022:4063315. doi: 10.1155/2022/4063315 (PMC9637474; doi:10.1155/2022/4063315)
Supplement: Supplementary Materials — KEGG pathway enrichment analysis result of BYHWD by using BATMAN-TCM. [file 4063315.f1.pdf]

Supplementary Table

KEGG pathway enrichment analysis result of BYHWD (score>=20)			
（Adjusted P_value is the P_value after Benjamini-Hochberg multiple testing correction. Enrichment Ratio is computed as the raito of the proportion of the proteins belong to the term among BYHWD's targets to the proportion of the proteins belong to the term among the whole proteome）			
Category	Annotation Term ID	Term description	p-value|Adjusted P_value|the number of targets mapped to this term|Enrichment Ratio|target list mapped to this term
KEGG	#	Endocrine system	1.23e-003|6.81e-003|90|1.3|ABCC8;ACADM;ACOX1;ACSL1;AKR1C3;ALOX5;APOA1;APOA2;ARAF;ATP1A1;<br>BAD;BMP4;CACNA1C;CACNA1D;CACNA2D1;CACNA2D2;CAMK2D;CAMK2G;CDC42;CDKN1A;<br>CREB1;CTNNB1;CYP11A1;CYP19A1;CYP1A1;CYP27A1;DCT;EDN1;ESR1;FABP2;<br>FABP6;FADS2;FBP1;GNRH1;GPX7;GSR;GUCY1B3;HDAC1;HDAC2;HIF1A;<br>HSD17B1;HSD17B2;HSP90AA1;HSP90AB1;IGF1;INSR;IYD;KCNMA1;LEP;MAP2K5;<br>MED1;MMP9;NCOA1;NOS3;NPR1;NPR2;OXT;OXTR;PDE3A;PDE3B;<br>PGR;PLCG2;PLD1;PLD2;PLN;PPARD;PPARG;PRKAA1;PRKAB1;PRKAB2;<br>PRKCD;PRLR;PTGS2;PYGL;RAB3A;ROCK1;ROCK2;RXRA;RYR2;RYR3;<br>STAR;STX1A;TH;TPO;TYR;TYRP1;WNT10B;WNT2;WNT2B;WNT4;<br>|
KEGG	#	Signal transduction	3.75e-001|7.47e-001|150|1.0|ABL1;ABL2;ACVR1;ACVR1B;ADORA1;ADORA2A;ADORA2B;ADORA3;ADRA2A;ADRA2B;<br>ADRA2C;AMHR2;ANGPT1;ARAF;ATP1A1;BAD;BCL2;BCL2L11;BDKRB2;BMP4;<br>BNIP3;BRCA1;CACNA1A;CACNA1B;CACNA1C;CACNA1D;CACNA2D1;CACNA2D2;CAMK2D;CAMK2G;<br>CAT;CCL2;CCL5;CDC42;CDKN1A;CFTR;CNR1;CREB1;CTNNB1;DGKA;<br>DGKI;DLG4;DRD1;DRD2;DRD5;EDN1;EGLN1;EGLN2;EGLN3;EPO;<br>FADD;FAS;FASLG;FBP1;FGF10;FGF23;FGFR2;GRIN1;GRIN2A;GRIN2B;<br>GRIN2C;GRIN2D;GUCY1B3;HDAC1;HDAC2;HIF1A;HMOX1;HSP90AA1;HSP90AB1;HTR2A;<br>HTR2B;HTR2C;HTR7;IFNG;IGF1;IL10;IL4;INSR;KCNMA1;LEP;<br>LTA;MAGI2;MAP2K5;MMP9;NFKB2;NGFR;NOD2;NOS1;NOS2;NOS3;<br>NPPA;NPR1;NPR2;NRG1;OXTR;P2RX2;P2RX3;PDE1B;PDE2A;PDE3A;<br>PDE3B;PDE5A;PIM1;PLA2G1B;PLCG2;PLD1;PLD2;PLN;PPARD;PPARG;<br>PPP2CA;PPP2CB;PRKAA1;PRKAB1;PRKAB2;PRLR;PTGER1;PTGER3;PTGS2;RAP1GAP;<br>RAPGEF2;RIPK1;ROCK1;ROCK2;RXRA;RYR2;RYR3;SHH;SIRT1;SLC25A4;<br>SLC8A1;SMAD7;SMO;SNAI2;SNW1;STIM1;STIM2;TACR2;TGFB2;TIRAP;<br>TNNC1;TPO;USP7;VDAC1;VDAC2;VDAC3;WNT10B;WNT2;WNT2B;WNT4;<br>|
KEGG	#	Overview	5.20e-015|2.95e-013|53|3.1|ACADM;ACADS;ACADSB;ACAT1;ACO2;ACOX1;ACSL1;ACSS1;ACSS2;ACY1;<br>AGXT;ALDH7A1;ARG1;ARG2;ASL;ASS1;BCAT1;BCAT2;CBS;CPS1;<br>DLD;DLST;FADS1;FADS2;FBP1;GLDC;GLUL;GOT1;GOT2;G

			PT;<br>GPT2;MAT1A;MAT2A;MTHFR;MTR;MUT;NAGS;OGDH;OGDHL;OTC;<br>PAH;PCCB;PHGDH;PRPS1;SDHA;SDHB;SDHC;SDHD;SHMT1;SHMT2;<br>SUCLA2;SUCLG1;SUCLG2;|
KEGG	#	Sensory system	1.00e+000|1.00e+000|20|0.4|ADRBK2;BDKRB2;CACNA1A;CACNA1B;CAMK2D;CAMK2G;F2RL1;HTR2A;HTR2B;HTR2C;<br>IGF1;KCNB1;PLCG2;PRKCD;PTGER2;PTGER4;TRPA1;TRPM8;TRPV1;TRPV3;<br>|
KEGG	#	Transcription	1.00e+000|1.00e+000|3|0.2|SNW1;TAF7;TBPL1;|
KEGG	#	Signaling molecules and interaction	1.62e-003|7.99e-003|97|1.3|ACVR1;ACVR1B;ADORA1;ADORA2A;ADORA2B;ADORA3;ADRA2A;ADRA2B;ADRA2C;AGRN;<br>AMHR2;BDKRB2;CCL2;CCL5;CDH3;CHRNA3;CHRNB2;CHRNB4;CNR1;CNR2;<br>CRHR2;CX3CR1;CXCR4;DRD1;DRD2;DRD3;DRD4;DRD5;EDA;EPO;<br>F2RL1;FAS;FASLG;GABRA1;GABRA2;GABRA3;GABRA4;GABRA5;GABRA6;GABRB1;<br>GABRB2;GABRB3;GABRD;GABRE;GABRG1;GABRG2;GABRG3;GABRP;GABRQ;GLRA1;<br>GLRA2;GLRA3;GLRB;GRIN1;GRIN2A;GRIN2B;GRIN2C;GRIN2D;GRIN3A;GRIN3B;<br>HTR1A;HTR1B;HTR1D;HTR2A;HTR2B;HTR2C;HTR7;IFNG;IL10;IL4;<br>LEP;LRRC4B;LTA;MC4R;MLNR;NGFR;NLGN1;NPY2R;NR3C1;NRXN1;<br>NRXN2;NRXN3;OPRK1;OXTR;P2RX2;P2RX3;PF4;PLG;PRLR;PTGER1;<br>PTGER2;PTGER3;PTGER4;TACR2;TGFB2;TPO;TRPV1;|
KEGG	#	Membrane transport	6.08e-002|1.94e-001|8|1.9|ABCA1;ABCB1;ABCB11;ABCC2;ABCC8;ABCC9;ABCG1;CFTR;|
KEGG	#	Glycan biosynthesis and metabolism	1.00e+000|1.00e+000|6|0.3|B4GALT1;COLGALT1;COLGALT2;GPLD1;HYAL2;PLOD3;|
KEGG	#	Biosynthesis of other secondary metabolites	2.56e-001|5.59e-001|2|2.0|CYP1A2;XDH;|
KEGG	#	Development	9.81e-001|1.00e+000|17|0.7|ABL1;CDC42;CREB1;CXCR4;CYBA;EPHA4;IFNG;LILRB1;NFKB2;PLCG2;<br>PPARG;ROCK1;ROCK2;SEMA4D;SLIT2;SPI1;TGFB2;|
KEGG	#	Folding, sorting and degradation	1.00e+000|1.00e+000|19|0.4|BAK1;BAX;BCL2;BRCA1;EIF2AK1;ERO1B;HSP90AA1;HSP90AB1;IFNG;NEDD4;<br>NEDD4L;NFS1;P4HB;PML;STUB1;STX1A;TST;UBE2B;VCP;|
KEGG	#	Energy metabolism	3.56e-002|1.30e-001|23|1.4|COX1;COX2;COX3;COX4I1;COX5A;COX5B;COX6A2;COX6B1;COX6C;COX7A1;<br>COX7B;COX7C;COX8A;CPS1;ETHE1;GLUL;SDHA;SDHB;SDHC;SDHD;<br>SQRDL;SUOX;TST;|

<table>
<tr><td>KEGG</td><td>#</td><td>Amino acid metabolism</td><td>4.36e-055|9.90e-053|123|4.6|ABAT;ACAD8;ACADM;ACADS;ACADSB;ACAT1;ACY1;ACY3;ADH1A;ADH1B;
ADH1C;ADH4;ADH7;ADSL;ADSS;ADSSL1;AGMAT;AGXT;AGXT2;AHCY;
ALAS1;ALAS2;ALDH1B1;ALDH2;ALDH3B1;ALDH3B2;ALDH5A1;ALDH7A1;ALDH9A1;AMD1;
ARG1;ARG2;ASL;ASNS;ASPA;ASS1;AZIN2;BBOX1;BCAT1;BCAT2;
BCKDHA;BCKDHB;CAD;CAT;CBS;CHDH;COLGALT1;COLGALT2;COMT;CPS1;
CYP1A1;CYP1A2;DAO;DBH;DBT;DCT;DDC;DLD;DLST;DMGDH;
DNMT1;DNMT3A;DNMT3B;GAMT;GATM;GCAT;GCDH;GCSH;GLDC;GLUL;
GLYAT;GNMT;GOT1;GOT2;GPT;GPT2;HPD;IDO1;IL4I1;IVD;
KMT2A;KYNU;MAOA;MAOB;MAT1A;MAT2A;MIF;MTAP;MTR;MUT;
NAGS;NOS1;NOS2;NOS3;OAT;ODC1;OGDH;OGDHL;OTC;OXCT1;
OXCT2;P4HA1;P4HA2;P4HA3;PAH;PCCB;PHGDH;PHYKPL;PIPOX;PLOD1;
PLOD2;PLOD3;PPAT;SHMT1;SHMT2;TH;TMLHE;TPH1;TPH2;TPO;
TST;TYR;TYRP1;|</td></tr>
<tr><td>KEGG</td><td>#</td><td>Cell motility</td><td>9.98e-001|1.00e+000|10|0.5|ACTN3;ARAF;BDKRB2;CDC42;FGF10;FGF23;FGFR2;NCKAP1L;ROCK1;ROCK2;
|</td></tr>
<tr><td>KEGG</td><td>#</td><td>Excretory system</td><td>4.19e-001|7.92e-001|16|1.1|AQP1;ATP1A1;BDKRB2;CALB1;CREB1;DNM3;ESR1;IGF1;INSR;KL;
NEDD4L;NR3C2;SLC25A10;SLC38A3;SLC8A1;VDR;|</td></tr>
<tr><td>KEGG</td><td>#</td><td>Metabolism of terpenoids and polyketides</td><td>6.24e-001|1.00e+000|2|1.0|ACAT1;FNTA;|</td></tr>
<tr><td>KEGG</td><td>#</td><td>Digestive system</td><td>3.66e-002|1.30e-001|48|1.3|ABCA1;ABCB1;ABCB11;ABCC2;APOA1;AQP1;AQP8;ATP1A1;BAAT;CACNA1D;
CAMK2D;CAMK2G;CFTR;CUBN;CYP3A4;DGAT2;FABP2;GOT2;GUCY1B3;HMOX1;
KCNMA1;KCNQ1;LYZ;MMACHC;MTTP;NOS1;NR1H4;PLA2G1B;PNLIPRP2;RXRA;
RYR2;RYR3;S100G;SLC11A1;SLC11A2;SLC1A1;SLC1A5;SLC36A1;SLC3A1;SLC5A6;
SLC7A7;SLC7A8;SLC8A1;SLCO1B1;SLCO1B3;SST;SULT2A1;VDR;|</td></tr>
<tr><td>KEGG</td><td>#</td><td>Transport and catabolism</td><td>9.98e-001|1.00e+000|37|0.7|ACOX1;ACSL1;ADRBK1;ADRBK2;AGXT;AP3D1;BAAT;CAT;CAV3;CDC42;
CLN3;CXCR4;CYBA;DAO;DNM3;FGFR2;FOLR1;FOLR2;GALC;HACL1;
IFNG;NEDD4;NEDD4L;NOS1;NOS2;NUDT12;PAOX;PIPOX;PLD1;PLD2;
PML;PRKAA1;SLC11A1;SLC11A2;SMAD7;TGFB2;XDH;|</td></tr>
<tr><td>KEGG</td><td>#</td><td>Immune system</td><td>1.00e+000|1.00e+000|44|0.5|ACTN3;ADRBK1;ADRBK2;ARAF;BDKRB2;CCL2;CCL5;CD74;CDC42;CREB1;
CTNNB1;CX3CR1;CXCR4;CYBA;EPO;FADD;FAS;FASLG;FCER2;GUCY1B3;
HSP90AA1;HSP90AB1;IFNG;IL10;IL4;MMP9;NOD2;NOS3;PF4;PLAT;
PLCG2;PLD1;PLD2;PLG;PRKCD;PTGS1;RIPK1;ROCK1;ROCK2;STIM1;
THBD;TIRAP;TLR3;TPO;|</td></tr>
</table>

<table>
<tr><td>KEGG</td><td>#</td><td>Nucleotide metabolism</td><td>2.59e-013|9.80e-012|55|2.9|ADA;ADK;ADSL;ADSS;ADSSL1;AK5;APRT;ATIC;CAD;CMPK2;
CTPS1;DCK;DGUOK;DHODH;DPYD;DPYS;DTYMK;DUT;ENPP1;ENPP3;
GUCY1B3;GUK1;IMPDH1;IMPDH2;NPR1;NPR2;NT5C2;NT5M;PAICS;PDE10A;
PDE11A;PDE1B;PDE2A;PDE3A;PDE3B;PDE4A;PDE4B;PDE4D;PDE5A;PDE9A;
PNP;POLA1;POLD1;POLE;POLE2;POLE3;POLE4;PPAT;PRPS1;RRM1;
RRM2;RRM2B;TXNRD1;TYMS;XDH;|</td></tr>
<tr><td>KEGG</td><td>#</td><td>Circulatory system</td><td>1.95e-004|1.30e-003|44|1.7|ADORA2A;ADORA2B;ARAF;ATP1A1;BCL2;CACNA1C;CACNA1D;CACNA2D1;CACNA2D2;CAMK2D;
CAMK2G;COX1;COX2;COX3;COX4I1;COX5A;COX5B;COX6A2;COX6B1;COX6C;
COX7A1;COX7B;COX7C;COX8A;CREB1;GUCY1B3;KCNMA1;KCNQ1;NPR1;NPR2;
PLA2G1B;PLN;PPP2CA;PPP2CB;PRKCD;ROCK1;ROCK2;RYR2;SCN1B;SCN4B;
SCN5A;SCN7A;SLC8A1;TNNC1;|</td></tr>
<tr><td>KEGG</td><td>#</td><td>Cell growth and death</td><td>8.48e-001|1.00e+000|25|0.8|ABL1;AIFM1;APAF1;AR;AURKA;BAD;BAX;BCL2;CAMK2D;CAMK2G;
CDKN1A;FADD;FAS;FASLG;HDAC1;HDAC2;IGF1;PGR;PPP2CA;PPP2CB;
PRKDC;RIPK1;RRM2;RRM2B;TGFB2;|</td></tr>
<tr><td>KEGG</td><td>#</td><td>Cell communication</td><td>9.99e-001|1.00e+000|24|0.6|ACTN3;BAD;BCL2;CAV3;CDC42;CTNNB1;DRD1;DRD2;GUCY1B3;HTR2A;
HTR2B;HTR2C;IGF1;INSR;MAGI2;MAP2K5;PPP2CA;PPP2CB;PRKCD;ROCK1;
ROCK2;SNAI1;SNAI2;YBX3;|</td></tr>
<tr><td>KEGG</td><td>#</td><td>Nervous system</td><td>2.06e-014|9.35e-013|100|2.1|ABAT;ABL1;ACHE;ADRBK1;ADRBK2;ALOX5;ARAF;BAD;BAX;BCL2;
CACNA1A;CACNA1B;CACNA1C;CACNA1D;CALY;CAMK2D;CAMK2G;CDC42;CHRNA3;CHRNB2;
CHRNB4;CNR1;COMT;CREB1;CRH;DDC;DLG4;DNM3;DRD1;DRD2;
DRD3;DRD4;DRD5;FASLG;GABRA1;GABRA2;GABRA3;GABRA4;GABRA5;GABRA6;
GABRB1;GABRB2;GABRB3;GABRD;GABRE;GABRG1;GABRG2;GABRG3;GABRP;GABRQ;
GLUL;GRIN1;GRIN2A;GRIN2B;GRIN2C;GRIN2D;GRIN3A;GRIN3B;GUCY1B3;HAP1;
HTR1A;HTR1B;HTR1D;HTR2A;HTR2B;HTR2C;HTR7;IGF1;KCND2;KCNQ1;
MAOA;MAOB;MAP2K5;NAPEPLD;NGFR;NOS1;PLCG2;PLD1;PLD2;PPP2CA;
PPP2CB;PRKCD;PTGS1;PTGS2;RAB3A;SCN1A;SHANK3;SLC17A7;SLC1A1;SLC1A3;
SLC1A6;SLC32A1;SLC38A3;SLC5A7;SLC6A3;SLC6A4;STX1A;TH;TPH1;TPH2;
|</td></tr>
<tr><td>KEGG</td><td>#</td><td>Translation</td><td>1.00e+000|1.00e+000|18|0.4|AARS;AARS2;DARS;DARS2;GARS;IARS;IARS2;KARS;LARS;LARS2;
NARS;NARS2;PPP2CA;PPP2CB;RARS;TARS;TARS2;VARS;|</td></tr>
<tr><td>KEGG</td><td>#</td><td>Metabolism of other amino acids</td><td>2.44e-004|1.50e-003|23|2.1|ABAT;ACADM;ALDH1B1;ALDH2;ALDH3B1;ALDH3B2;ALDH7A</td></tr>
</table>

<table>
<tr><td></td><td></td><td></td><td>1;ALDH9A1;BAAT;DAO;
DPYD;DPYS;GPX7;GSR;GSS;MTR;ODC1;RRM1;RRM2;RRM2B;
SHMT1;SHMT2;TXNRD1;|</td></tr>
<tr><td>KEGG</td><td>#</td><td>Lipid metabolism</td><td>3.36e-010|6.36e-009|75|2.1|ACADM;ACADS;ACADSB;ACAT1;ACHE;ACOT4;ACOX1;ACSL1;ADH1A;ADH1B;
ADH1C;ADH4;ADH7;AKR1C2;AKR1C3;AKR1D1;ALDH1B1;ALDH2;ALDH7A1;ALDH9A1;
ALOX5;BAAT;BDH1;CERS1;CHKA;COMT;CYP11A1;CYP19A1;CYP1A1;CYP1A2;
CYP24A1;CYP27A1;CYP27B1;CYP2E1;CYP2R1;CYP39A1;CYP3A4;CYP51A1;DGAT2;DGKA;
DGKI;ELOVL4;ENPP6;FADS1;FADS2;GAL3ST1;GALC;GCDH;GPD1L;GPX7;
HSD17B1;HSD17B2;HSD17B6;HSD17B8;OXCT1;OXCT2;PCYT1A;PCYT1B;PHOSPHO1;PLA2G1B;
PLD1;PLD2;PLD3;PLD4;PNLIPRP2;PTGS1;PTGS2;SOAT1;SOAT2;SRD5A1;
SRD5A2;SRD5A3;UGCG;UGT1A1;UGT8;|</td></tr>
<tr><td>KEGG</td><td>#</td><td>Replication and repair</td><td>9.52e-001|1.00e+000|10|0.7|BRCA1;LIG4;POLA1;POLB;POLD1;POLE;POLE2;POLE3;POLE4;PRKDC;
|</td></tr>
<tr><td>KEGG</td><td>#</td><td>Metabolism of cofactors and vitamins</td><td>4.88e-008|6.52e-007|44|2.3|ADH1A;ADH1B;ADH1C;ADH4;ADH7;ALAS1;ALAS2;ATIC;BCAT1;BCAT2;
CYP1A1;CYP1A2;CYP3A4;DHFR;DHFRL1;DHRS9;DPYD;DPYS;ENPP1;ENPP3;
FECH;HMBS;HMOX1;HPD;HSD17B6;LIAS;LIPT1;MMAB;MTHFR;MTR;
NFS1;NQO1;NT5C2;NT5M;NUDT12;PNP;SHMT1;SHMT2;TYMS;TYR;
UGT1A1;UROD;UROS;VKORC1;|</td></tr>
<tr><td>KEGG</td><td>#</td><td>Carbohydrate metabolism</td><td>2.21e-004|1.43e-003|52|1.6|ABAT;ACADM;ACADS;ACAT1;ACO2;ACSS1;ACSS2;ADH1A;ADH1B;ADH1C;
ADH4;ADH7;AGXT;ALDH1B1;ALDH2;ALDH3B1;ALDH3B2;ALDH5A1;ALDH7A1;ALDH9A1;
B4GALT1;BDH1;CAT;CYB5R1;CYB5R3;DLD;DLST;ENPP1;ENPP3;FBP1;
GCSH;GLUL;KL;MUT;OGDH;OGDHL;OXCT1;OXCT2;PCCB;PLCG2;
PRPS1;PYGL;SDHA;SDHB;SDHC;SDHD;SHMT1;SHMT2;SUCLA2;SUCLG1;
SUCLG2;UGT1A1;|</td></tr>
<tr><td>KEGG</td><td>#</td><td>Xenobiotics biodegradation and metabolism</td><td>7.23e-004|4.10e-003|22|2.0|ADH1A;ADH1B;ADH1C;ADH4;ADH7;AKR1C2;ALDH3B1;ALDH3B2;CES1;CYP1A1;
CYP1A2;CYP2E1;CYP3A4;DPYD;DPYS;IMPDH1;IMPDH2;MAOA;MAOB;SULT2A1;
UGT1A1;XDH;|</td></tr>
<tr><td>KEGG</td><td>#</td><td>Environmental adaptation</td><td>8.02e-002|2.40e-001|17|1.4|CACNA1C;CACNA1D;CAMK2D;CAMK2G;CREB1;GRIN1;GRIN2A;GRIN2B;GRIN2C;GRIN2D;
GUCY1B3;NOS1;PRKAA1;PRKAB1;PRKAB2;RYR2;RYR3;|</td></tr>
<tr><td>KEGG</td><td>hsa00010</td><td>Glycolysis / Gluconeogenesis</td><td>1.44e-003|7.78e-003|15|2.3|ACSS1;ACSS2;ADH1A;ADH1B;ADH1C;ADH4;ADH7;ALDH1B1;ALDH2;ALDH3B1;
ALDH3B2;ALDH7A1;ALDH9A1;DLD;FBP1;|</td></tr>
</table>

<table>
<tr><td>KEGG</td><td>hsa00020</td><td>Citrate cycle (TCA cycle)</td><td>1.15e-005|9.00e-005|12|4.1|ACO2;DLD;DLST;OGDH;OGDHL;SDHA;SDHB;SDHC;SDHD;SUCLA2;
SUCLG1;SUCLG2;|</td></tr>
<tr><td>KEGG</td><td>hsa00030</td><td>Pentose phosphate pathway</td><td>7.75e-001|1.00e+000|2|0.7|FBP1;PRPS1;|</td></tr>
<tr><td>KEGG</td><td>hsa00040</td><td>Pentose and glucuronate interconversions</td><td>4.30e-001|8.00e-001|4|1.2|ALDH1B1;ALDH2;KL;UGT1A1;|</td></tr>
<tr><td>KEGG</td><td>hsa00051</td><td>Fructose and mannose metabolism</td><td>9.63e-001|1.00e+000|1|0.3|FBP1;|</td></tr>
<tr><td>KEGG</td><td>hsa00052</td><td>Galactose metabolism</td><td>9.59e-001|1.00e+000|1|0.3|B4GALT1;|</td></tr>
<tr><td>KEGG</td><td>hsa00053</td><td>Ascorbate and aldarate metabolism</td><td>1.18e-001|3.11e-001|5|1.9|ALDH1B1;ALDH2;ALDH7A1;ALDH9A1;UGT1A1;|</td></tr>
<tr><td>KEGG</td><td>hsa00062</td><td>Fatty acid elongation</td><td>6.74e-001|1.00e+000|2|0.9|ACOT4;ELOVL4;|</td></tr>
<tr><td>KEGG</td><td>hsa00071</td><td>Fatty acid degradation</td><td>1.79e-006|1.77e-005|16|3.7|ACADM;ACADS;ACADSB;ACAT1;ACOX1;ACSL1;ADH1A;ADH1B;ADH1C;ADH4;
ADH7;ALDH1B1;ALDH2;ALDH7A1;ALDH9A1;GCDH;|</td></tr>
<tr><td>KEGG</td><td>hsa00072</td><td>Synthesis and degradation of ketone bodies</td><td>7.68e-003|3.49e-002|4|4.5|ACAT1;BDH1;OXCT1;OXCT2;|</td></tr>
<tr><td>KEGG</td><td>hsa00100</td><td>Steroid biosynthesis</td><td>7.66e-003|3.49e-002|6|3.2|CYP24A1;CYP27B1;CYP2R1;CYP51A1;SOAT1;SOAT2;|</td></tr>
<tr><td>KEGG</td><td>hsa00120</td><td>Primary bile acid biosynthesis</td><td>7.74e-002|2.37e-001|4|2.4|AKR1D1;BAAT;CYP27A1;CYP39A1;|</td></tr>
<tr><td>KEGG</td><td>hsa00130</td><td>Ubiquinone and other terpenoid-quinone biosynthesis</td><td>6.65e-002|2.10e-001|3|3.1|HPD;NQO1;VKORC1;|</td></tr>
<tr><td>KEGG</td><td>hsa00140</td><td>Steroid hormone biosynthesis</td><td>4.26e-006|4.03e-005|18|3.2|AKR1C2;AKR1C3;AKR1D1;COMT;CYP11A1;CYP19A1;CYP1A1;CYP1A2;CYP2E1;CYP3A4;
HSD17B1;HSD17B2;HSD17B6;HSD17B8;SRD5A1;SRD5A2;SRD5A3;UGT1A1;|</td></tr>
</table>

<table>
<tr><td>KEGG</td><td>hsa00190</td><td>Oxidative phosphorylation</td><td>1.45e-001|3.70e-001|17|1.3|COX1;COX2;COX3;COX4I1;COX5A;COX5B;COX6A2;COX6B1;COX6C;COX7A1;
COX7B;COX7C;COX8A;SDHA;SDHB;SDHC;SDHD;|</td></tr>
<tr><td>KEGG</td><td>hsa00230</td><td>Purine metabolism</td><td>2.09e-010|4.31e-009|45|2.6|ADA;ADK;ADSL;ADSS;ADSSL1;AK5;APRT;ATIC;DCK;DGUOK;
ENPP1;ENPP3;GUCY1B3;GUK1;IMPDH1;IMPDH2;NPR1;NPR2;NT5C2;NT5M;
PAICS;PDE10A;PDE11A;PDE1B;PDE2A;PDE3A;PDE3B;PDE4A;PDE4B;PDE4D;
PDE5A;PDE9A;PNP;POLA1;POLD1;POLE;POLE2;POLE3;POLE4;PPAT;
PRPS1;RRM1;RRM2;RRM2B;XDH;|</td></tr>
<tr><td>KEGG</td><td>hsa00232</td><td>Caffeine metabolism</td><td>7.84e-002|2.37e-001|2|4.1|CYP1A2;XDH;|</td></tr>
<tr><td>KEGG</td><td>hsa00240</td><td>Pyrimidine metabolism</td><td>1.56e-004|1.07e-003|23|2.3|CAD;CMPK2;CTPS1;DCK;DHODH;DPYD;DPYS;DTYMK;DUT;NT5C2;
NT5M;PNP;POLA1;POLD1;POLE;POLE2;POLE3;POLE4;RRM1;RRM2;
RRM2B;TXNRD1;TYMS;|</td></tr>
<tr><td>KEGG</td><td>hsa00250</td><td>Alanine, aspartate and glutamate metabolism</td><td>6.26e-013|1.58e-011|21|6.0|ABAT;ACY3;ADSL;ADSS;ADSSL1;AGXT;AGXT2;ALDH5A1;ASL;ASNS;
ASPA;ASS1;CAD;CPS1;GLUL;GOT1;GOT2;GPT;GPT2;IL4I1;
PPAT;|</td></tr>
<tr><td>KEGG</td><td>hsa00260</td><td>Glycine, serine and threonine metabolism</td><td>4.49e-013|1.27e-011|22|5.8|AGXT;AGXT2;ALAS1;ALAS2;ALDH7A1;CBS;CHDH;DAO;DLD;DMGDH;
GAMT;GATM;GCAT;GCSH;GLDC;GNMT;MAOA;MAOB;PHGDH;PIPOX;
SHMT1;SHMT2;|</td></tr>
<tr><td>KEGG</td><td>hsa00270</td><td>Cysteine and methionine metabolism</td><td>4.62e-006|4.19e-005|14|3.9|AHCY;AMD1;CBS;DNMT1;DNMT3A;DNMT3B;GOT1;GOT2;IL4I1;MAT1A;
MAT2A;MTAP;MTR;TST;|</td></tr>
<tr><td>KEGG</td><td>hsa00280</td><td>Valine, leucine and isoleucine degradation</td><td>1.15e-011|2.61e-010|22|5.1|ABAT;ACAD8;ACADM;ACADS;ACADSB;ACAT1;ALDH1B1;ALDH2;ALDH7A1;ALDH9A1;
BCAT1;BCAT2;BCKDHA;BCKDHB;DBT;DLD;IL4I1;IVD;MUT;OXCT1;
OXCT2;PCCB;|</td></tr>
<tr><td>KEGG</td><td>hsa00290</td><td>Valine, leucine and isoleucine biosynthesis</td><td>5.02e-002|1.68e-001|2|5.1|BCAT1;BCAT2;|</td></tr>
<tr><td>KEGG</td><td>hsa00300</td><td>Lysine biosynthesis</td><td>1.86e-001|4.54e-001|1|5.0|ALDH7A1;|</td></tr>
<tr><td>KEGG</td><td>hsa00310</td><td>Lysine degradation</td><td>1.22e-007|1.54e-006|19|3.8|ACAT1;ALDH1B1;ALDH2;ALDH7A1;ALDH9A1;BBOX1;COLGALT1;COLGALT2;DLST;GCDH;
KMT2A;OGDH;OGDHL;PHYKPL;PIPOX;PLOD1;PLOD2;PLOD3;TMLHE;|</td></tr>
</table>

KEGG	hsa00330	Arginine and proline metabolism	9.90e-017|1.12e-014|31|5.3|ACY1;AGMAT;ALDH1B1;ALDH2;ALDH7A1;ALDH9A1;AMD1;ARG1;ARG2;ASL;<br>ASS1;AZIN2;CPS1;DAO;GAMT;GATM;GLUL;GOT1;GOT2;MAOA;<br>MAOB;NAGS;NOS1;NOS2;NOS3;OAT;ODC1;OTC;P4HA1;P4HA2;<br>P4HA3;|
KEGG	hsa00340	Histidine metabolism	3.28e-005|2.48e-004|11|4.1|ACY3;ALDH1B1;ALDH2;ALDH3B1;ALDH3B2;ALDH7A1;ALDH9A1;ASPA;DDC;MAOA;<br>MAOB;|
KEGG	hsa00350	Tyrosine metabolism	4.49e-013|1.27e-011|22|5.8|ADH1A;ADH1B;ADH1C;ADH4;ADH7;ALDH3B1;ALDH3B2;COMT;DBH;DCT;<br>DDC;GOT1;GOT2;HPD;IL4I1;MAOA;MAOB;MIF;TH;TPO;<br>TYR;TYRP1;|
KEGG	hsa00360	Phenylalanine metabolism	7.47e-009|1.06e-007|12|6.7|ALDH3B1;ALDH3B2;DDC;GLYAT;GOT1;GOT2;HPD;IL4I1;MAOA;MAOB;<br>MIF;PAH;|
KEGG	hsa00380	Tryptophan metabolism	9.30e-010|1.62e-008|19|4.9|ACAT1;ALDH1B1;ALDH2;ALDH7A1;ALDH9A1;CAT;CYP1A1;CYP1A2;DDC;GCDH;<br>IDO1;IL4I1;KYNU;MAOA;MAOB;OGDH;OGDHL;TPH1;TPH2;|
KEGG	hsa00400	Phenylalanine, tyrosine and tryptophan biosynthesis	4.20e-004|2.44e-003|4|8.2|GOT1;GOT2;IL4I1;PAH;|
KEGG	hsa00410	beta-Alanine metabolism	3.68e-004|2.20e-003|10|3.5|ABAT;ACADM;ALDH1B1;ALDH2;ALDH3B1;ALDH3B2;ALDH7A1;ALDH9A1;DPYD;DPYS;<br>|
KEGG	hsa00430	Taurine and hypotaurine metabolism	6.43e-001|1.00e+000|1|1.0|BAAT;|
KEGG	hsa00450	Selenocompound metabolism	5.07e-001|8.79e-001|2|1.2|MTR;TXNRD1;|
KEGG	hsa00460	Cyanoamino acid metabolism	1.44e-001|3.70e-001|2|2.9|SHMT1;SHMT2;|
KEGG	hsa00472	D-Arginine and D-ornithine metabolism	9.79e-002|2.68e-001|1|10.2|DAO;|
KEGG	hsa00480	Glutathione metabolism	2.28e-001|5.17e-001|7|1.4|GPX7;GSR;GSS;ODC1;RRM1;RRM2;RRM2B;|

KEGG	hsa00500	Starch and sucrose metabolism	6.53e-001|1.00e+000|5|0.9|ENPP1;ENPP3;KL;PYGL;UGT1A1;|
KEGG	hsa00510	N-Glycan biosynthesis	9.94e-001|1.00e+000|1|0.2|B4GALT1;|
KEGG	hsa00514	Other types of O-glycan biosynthesis	3.61e-001|7.32e-001|4|1.3|B4GALT1;COLGALT1;COLGALT2;PLOD3;|
KEGG	hsa00520	Amino sugar and nucleotide sugar metabolism	9.53e-001|1.00e+000|2|0.4|CYB5R1;CYB5R3;|
KEGG	hsa00531	Glycosaminoglycan degradation	8.59e-001|1.00e+000|1|0.5|HYAL2;|
KEGG	hsa00533	Glycosaminoglycan biosynthesis - keratan sulfate	7.87e-001|1.00e+000|1|0.7|B4GALT1;|
KEGG	hsa00561	Glycerolipid metabolism	1.54e-001|3.88e-001|8|1.5|ALDH1B1;ALDH2;ALDH7A1;ALDH9A1;DGAT2;DGKA;DGKI;PNLIPRP2;|
KEGG	hsa00562	Inositol phosphate metabolism	9.98e-001|1.00e+000|1|0.2|PLCG2;|
KEGG	hsa00563	Glycosylphosphatidylinositol(GPI)-anchor biosynthesis	9.24e-001|1.00e+000|1|0.4|GPLD1;|
KEGG	hsa00564	Glycerophospholipid metabolism	1.05e-001|2.80e-001|13|1.5|ACHE;CHKA;DGKA;DGKI;GPD1L;PCYT1A;PCYT1B;PHOSPHO1;PLA2G1B;PLD1;<br>PLD2;PLD3;PLD4;|
KEGG	hsa00565	Ether lipid metabolism	2.08e-001|4.92e-001|6|1.5|ENPP6;PLA2G1B;PLD1;PLD2;PLD3;PLD4;|
KEGG	hsa00590	Arachidonic acid metabolism	4.22e-001|7.92e-001|7|1.1|AKR1C3;ALOX5;CYP2E1;GPX7;PLA2G1B;PTGS1;PTGS2;|
KEGG	hsa00591	Linoleic acid metabolism	2.92e-001|6.19e-001|4|1.5|CYP1A2;CYP2E1;CYP3A4;PLA2G1B;|
KEGG	hsa00592	alpha-Linolenic acid metabolism	4.22e-001|7.92e-001|3|1.3|ACOX1;FADS2;PLA2G1B;|

<table>
<tr><td>KEGG</td><td>hsa00600</td><td>Sphingolipid metabolism</td><td>4.93e-001|8.79e-001|5|1.1|CERS1;GAL3ST1;GALC;UGCG;UGT8;|</td></tr>
<tr><td>KEGG</td><td>hsa00601</td><td>Glycosphingolipid biosynthesis - lacto and neolacto series</td><td>9.32e-001|1.00e+000|1|0.4|B4GALT1;|</td></tr>
<tr><td>KEGG</td><td>hsa00620</td><td>Pyruvate metabolism</td><td>4.80e-002|1.65e-001|8|2.0|ACAT1;ACSS1;ACSS2;ALDH1B1;ALDH2;ALDH7A1;ALDH9A1;DLD;|</td></tr>
<tr><td>KEGG</td><td>hsa00630</td><td>Glyoxylate and dicarboxylate metabolism</td><td>6.29e-005|4.61e-004|10|4.2|ACAT1;ACO2;AGXT;CAT;GCSH;GLUL;MUT;PCCB;SHMT1;SHMT2;
|</td></tr>
<tr><td>KEGG</td><td>hsa00640</td><td>Propanoate metabolism</td><td>5.68e-007|6.45e-006|14|4.5|ABAT;ACADM;ACAT1;ACSS1;ACSS2;ALDH1B1;ALDH2;ALDH7A1;ALDH9A1;MUT;
PCCB;SUCLA2;SUCLG1;SUCLG2;|</td></tr>
<tr><td>KEGG</td><td>hsa00650</td><td>Butanoate metabolism</td><td>1.05e-002|4.50e-002|7|2.8|ABAT;ACADS;ACAT1;ALDH5A1;BDH1;OXCT1;OXCT2;|</td></tr>
<tr><td>KEGG</td><td>hsa00670</td><td>One carbon pool by folate</td><td>2.29e-004|1.44e-003|8|4.2|ATIC;DHFR;DHFRL1;MTHFR;MTR;SHMT1;SHMT2;TYMS;|</td></tr>
<tr><td>KEGG</td><td>hsa00730</td><td>Thiamine metabolism</td><td>3.38e-001|6.91e-001|1|2.6|NFS1;|</td></tr>
<tr><td>KEGG</td><td>hsa00740</td><td>Riboflavin metabolism</td><td>1.05e-001|2.80e-001|3|2.5|ENPP1;ENPP3;TYR;|</td></tr>
<tr><td>KEGG</td><td>hsa00760</td><td>Nicotinate and nicotinamide metabolism</td><td>5.01e-002|1.68e-001|6|2.2|ENPP1;ENPP3;NT5C2;NT5M;NUDT12;PNP;|</td></tr>
<tr><td>KEGG</td><td>hsa00770</td><td>Pantothenate and CoA biosynthesis</td><td>4.14e-003|2.00e-002|6|3.5|BCAT1;BCAT2;DPYD;DPYS;ENPP1;ENPP3;|</td></tr>
<tr><td>KEGG</td><td>hsa00785</td><td>Lipoic acid metabolism</td><td>2.69e-002|1.05e-001|2|6.9|LIAS;LIPT1;|</td></tr>
<tr><td>KEGG</td><td>hsa00790</td><td>Folate biosynthesis</td><td>4.05e-001|7.86e-001|2|1.4|DHFR;DHFRL1;|</td></tr>
<tr><td>KEGG</td><td>hsa00830</td><td>Retinol metabolism</td><td>3.63e-002|1.30e-001|11|1.8|ADH1A;ADH1B;ADH1C;ADH4;ADH7;CYP1A1;CYP1A2;CYP3A4;DHRS9;HSD17B6;
UGT1A1;|</td></tr>
</table>

<table>
<tr><td>KEGG</td><td>hsa00860</td><td>Porphyrin and chlorophyll metabolism</td><td>2.11e-002|8.40e-002|9|2.1|ALAS1;ALAS2;FECH;HMBS;HMOX1;MMAB;UGT1A1;UROD;UROS;|</td></tr>
<tr><td>KEGG</td><td>hsa00900</td><td>Terpenoid backbone biosynthesis</td><td>6.24e-001|1.00e+000|2|1.0|ACAT1;FNTA;|</td></tr>
<tr><td>KEGG</td><td>hsa00910</td><td>Nitrogen metabolism</td><td>5.07e-001|8.79e-001|2|1.2|CPS1;GLUL;|</td></tr>
<tr><td>KEGG</td><td>hsa00920</td><td>Sulfur metabolism</td><td>1.18e-002|4.87e-002|4|4.1|ETHE1;SQRDL;SUOX;TST;|</td></tr>
<tr><td>KEGG</td><td>hsa00970</td><td>Aminoacyl-tRNA biosynthesis</td><td>1.79e-006|1.77e-005|16|3.7|AARS;AARS2;DARS;DARS2;GARS;IARS;IARS2;KARS;LARS;LARS2;
NARS;NARS2;RARS;TARS;TARS2;VARS;|</td></tr>
<tr><td>KEGG</td><td>hsa00980</td><td>Metabolism of xenobiotics by cytochrome P450</td><td>1.15e-002|4.83e-002|14|1.9|ADH1A;ADH1B;ADH1C;ADH4;ADH7;AKR1C2;ALDH3B1;ALDH3B2;CYP1A1;CYP1A2;
CYP2E1;CYP3A4;SULT2A1;UGT1A1;|</td></tr>
<tr><td>KEGG</td><td>hsa00982</td><td>Drug metabolism - cytochrome P450</td><td>1.34e-002|5.43e-002|13|1.9|ADH1A;ADH1B;ADH1C;ADH4;ADH7;ALDH3B1;ALDH3B2;CYP1A2;CYP2E1;CYP3A4;
MAOA;MAOB;UGT1A1;|</td></tr>
<tr><td>KEGG</td><td>hsa00983</td><td>Drug metabolism - other enzymes</td><td>7.55e-002|2.35e-001|8|1.8|CES1;CYP3A4;DPYD;DPYS;IMPDH1;IMPDH2;UGT1A1;XDH;|</td></tr>
<tr><td>KEGG</td><td>hsa01040</td><td>Biosynthesis of unsaturated fatty acids</td><td>4.80e-002|1.65e-001|5|2.4|ACOT4;ACOX1;BAAT;FADS1;FADS2;|</td></tr>
<tr><td>KEGG</td><td>hsa01200</td><td>Carbon metabolism</td><td>2.79e-009|4.22e-008|32|3.2|ACADM;ACADS;ACAT1;ACO2;ACSS1;ACSS2;AGXT;CPS1;DLD;DLST;
FBP1;GLDC;GOT1;GOT2;GPT;GPT2;MTHFR;MUT;OGDH;OGDHL;
PCCB;PHGDH;PRPS1;SDHA;SDHB;SDHC;SDHD;SHMT1;SHMT2;SUCLA2;
SUCLG1;SUCLG2;|</td></tr>
<tr><td>KEGG</td><td>hsa01210</td><td>2-Oxocarboxylic acid metabolism</td><td>9.22e-006|7.83e-005|9|5.3|ACO2;ACY1;BCAT1;BCAT2;GOT1;GOT2;GPT;GPT2;NAGS;|</td></tr>
<tr><td>KEGG</td><td>hsa01212</td><td>Fatty acid metabolism</td><td>9.22e-002|2.58e-001|8|1.7|ACADM;ACADS;ACADSB;ACAT1;ACOX1;ACSL1;FADS1;FADS2;|</td></tr>
<tr><td>KEGG</td><td>hsa01230</td><td>Biosynthesis of amino acids</td><td>1.74e-009|2.82e-008|26|3.7|ACO2;ACY1;ALDH7A1;ARG1;ARG2;ASL;ASS1;BCAT1;BCAT2;CBS;
CPS1;GLUL;GOT1;GOT2;GPT;GPT2;MAT1A;MAT2A;MTR;NAGS;
OTC;PAH;PHGDH;PRPS1;SHMT1;SHMT2;|</td></tr>
</table>

KEGG	hsa02010	ABC transporters	6.08e-002|1.94e-001|8|1.9|ABCA1;ABCB1;ABCB11;ABCC2;ABCC8;ABCC9;ABCG1;CFTR;|
KEGG	hsa03015	mRNA surveillance pathway	9.99e-001|1.00e+000|2|0.2|PPP2CA;PPP2CB;|
KEGG	hsa03022	Basal transcription factors	9.48e-001|1.00e+000|2|0.4|TAF7;TBPL1;|
KEGG	hsa03030	DNA replication	1.35e-001|3.52e-001|6|1.7|POLA1;POLD1;POLE;POLE2;POLE3;POLE4;|
KEGG	hsa03040	Spliceosome	1.00e+000|1.00e+000|1|0.1|SNW1;|
KEGG	hsa03050	Proteasome	9.89e-001|1.00e+000|1|0.2|IFNG;|
KEGG	hsa03320	PPAR signaling pathway	3.40e-002|1.27e-001|12|1.8|ACADM;ACOX1;ACSL1;APOA1;APOA2;CYP27A1;FABP2;FABP6;FADS2;PPARD;<br>PPARG;RXRA;|
KEGG	hsa03410	Base excision repair	9.77e-002|2.68e-001|6|1.9|POLB;POLD1;POLE;POLE2;POLE3;POLE4;|
KEGG	hsa03420	Nucleotide excision repair	4.74e-001|8.61e-001|5|1.1|POLD1;POLE;POLE2;POLE3;POLE4;|
KEGG	hsa03430	Mismatch repair	9.07e-001|1.00e+000|1|0.4|POLD1;|
KEGG	hsa03440	Homologous recombination	9.45e-001|1.00e+000|1|0.4|POLD1;|
KEGG	hsa03450	Non-homologous end-joining	3.69e-001|7.41e-001|2|1.5|LIG4;PRKDC;|
KEGG	hsa03460	Fanconi anemia pathway	9.96e-001|1.00e+000|1|0.2|BRCA1;|
KEGG	hsa04010	MAPK signaling pathway	9.85e-001|1.00e+000|16|0.6|CACNA1A;CACNA1B;CACNA1C;CACNA1D;CACNA2D1;CACNA2D2;CDC42;FAS;FASLG;FGF10;<br>FGF23;FGFR2;MAP2K5;NFKB2;RAPGEF2;TGFB2;|
KEGG	hsa04012	ErbB signaling pathway	4.84e-001|8.72e-001|9|1.1|ABL1;ABL2;ARAF;BAD;CAMK2D;CAMK2G;CDKN1A;NRG1;PLCG2;|
KEGG	hsa04014	Ras signaling pathway	7.12e-001|1.00e+000|20|0.9|ABL1;ABL2;ANGPT1;BAD;CDC42;FASLG;FGF10;FGF2

			3;FGFR2;GRIN1;<br>GRIN2A;GRIN2B;HTR7;IGF1;INSR;NGFR;PLA2G1B;PLCG2;PLD1;PLD2;<br>|
KEGG	hsa04015	Rap1 signaling pathway	7.03e-001|1.00e+000|19|0.9|ADORA2A;ADORA2B;ANGPT1;CDC42;CNR1;CTNNB1;DRD2;FGF10;FGF23;FGFR2;<br>GRIN1;GRIN2A;GRIN2B;IGF1;INSR;MAGI2;NGFR;RAP1GAP;RAPGEF2;|
KEGG	hsa04020	Calcium signaling pathway	1.46e-007|1.74e-006|41|2.3|ADORA2A;ADORA2B;BDKRB2;CACNA1A;CACNA1B;CACNA1C;CACNA1D;CAMK2D;CAMK2G;DRD1;<br>DRD5;GRIN1;GRIN2A;GRIN2C;GRIN2D;HTR2A;HTR2B;HTR2C;HTR7;NOS1;<br>NOS2;NOS3;OXTR;P2RX2;P2RX3;PDE1B;PLCG2;PLN;PTGER1;PTGER3;<br>RYR2;RYR3;SLC25A4;SLC8A1;STIM1;STIM2;TACR2;TNNC1;VDAC1;VDAC2;<br>VDAC3;|
KEGG	hsa04022	cGMP - PKG signaling pathway	1.48e-003|7.81e-003|29|1.8|ADORA1;ADORA3;ADRA2A;ADRA2B;ADRA2C;ATP1A1;BAD;BDKRB2;CACNA1C;CACNA1D;<br>CREB1;GUCY1B3;INSR;KCNMA1;NOS3;NPR1;NPR2;PDE2A;PDE3A;PDE3B;<br>PDE5A;PLN;ROCK1;ROCK2;SLC25A4;SLC8A1;VDAC1;VDAC2;VDAC3;|
KEGG	hsa04060	Cytokine-cytokine receptor interaction	8.77e-001|1.00e+000|21|0.8|ACVR1;ACVR1B;AMHR2;CCL2;CCL5;CX3CR1;CXCR4;EDA;EPO;FAS;<br>FASLG;IFNG;IL10;IL4;LEP;LTA;NGFR;PF4;PRLR;TGFB2;<br>TPO;|
KEGG	hsa04062	Chemokine signaling pathway	9.82e-001|1.00e+000|11|0.6|ADRBK1;ADRBK2;CCL2;CCL5;CDC42;CX3CR1;CXCR4;PF4;PRKCD;ROCK1;<br>ROCK2;|
KEGG	hsa04064	NF-kappa B signaling pathway	8.01e-001|1.00e+000|7|0.8|BCL2;LTA;NFKB2;PLCG2;PTGS2;RIPK1;TIRAP;|
KEGG	hsa04066	HIF-1 signaling pathway	6.52e-003|3.08e-002|19|1.9|ANGPT1;BCL2;CAMK2D;CAMK2G;CDKN1A;EDN1;EGLN1;EGLN2;EGLN3;EPO;<br>HIF1A;HMOX1;IFNG;IGF1;INSR;NOS2;NOS3;NPPA;PLCG2;|
KEGG	hsa04068	FoxO signaling pathway	3.10e-001|6.52e-001|15|1.1|ARAF;BCL2L11;BNIP3;CAT;CDKN1A;FASLG;IGF1;IL10;INSR;PRKAA1;<br>PRKAB1;PRKAB2;SIRT1;TGFB2;USP7;|
KEGG	hsa04070	Phosphatidylinositol signaling system	9.88e-001|1.00e+000|3|0.4|DGKA;DGKI;PLCG2;|

<table>
<tr><td>KEGG</td><td>hsa04080</td><td>Neuroactive ligand-receptor interaction</td><td>3.17e-015|2.40e-013|71|2.6|ADORA1;ADORA2A;ADORA2B;ADORA3;ADRA2A;ADRA2B;ADRA2C;BDKRB2;CHRNA3;CHRNB2;
CHRNB4;CNR1;CNR2;CRHR2;DRD1;DRD2;DRD3;DRD4;DRD5;F2RL1;
GABRA1;GABRA2;GABRA3;GABRA4;GABRA5;GABRA6;GABRB1;GABRB2;GABRB3;GABRD;
GABRE;GABRG1;GABRG2;GABRG3;GABRP;GABRQ;GLRA1;GLRA2;GLRA3;GLRB;
GRIN1;GRIN2A;GRIN2B;GRIN2C;GRIN2D;GRIN3A;GRIN3B;HTR1A;HTR1B;HTR1D;
HTR2A;HTR2B;HTR2C;HTR7;LEP;MC4R;MLNR;NPY2R;NR3C1;OPRK1;
OXTR;P2RX2;P2RX3;PLG;PRLR;PTGER1;PTGER2;PTGER3;PTGER4;TACR2;
TRPV1;|</td></tr>
<tr><td>KEGG</td><td>hsa04110</td><td>Cell cycle</td><td>9.86e-001|1.00e+000|6|0.5|ABL1;CDKN1A;HDAC1;HDAC2;PRKDC;TGFB2;|</td></tr>
<tr><td>KEGG</td><td>hsa04114</td><td>Oocyte meiosis</td><td>8.57e-001|1.00e+000|8|0.7|AR;AURKA;CAMK2D;CAMK2G;IGF1;PGR;PPP2CA;PPP2CB;|</td></tr>
<tr><td>KEGG</td><td>hsa04115</td><td>p53 signaling pathway</td><td>5.04e-001|8.79e-001|7|1.0|APAF1;BAX;CDKN1A;FAS;IGF1;RRM2;RRM2B;|</td></tr>
<tr><td>KEGG</td><td>hsa04120</td><td>Ubiquitin mediated proteolysis</td><td>9.94e-001|1.00e+000|6|0.5|BRCA1;NEDD4;NEDD4L;PML;STUB1;UBE2B;|</td></tr>
<tr><td>KEGG</td><td>hsa04122</td><td>Sulfur relay system</td><td>2.56e-001|5.59e-001|2|2.0|NFS1;TST;|</td></tr>
<tr><td>KEGG</td><td>hsa04130</td><td>SNARE interactions in vesicular transport</td><td>9.70e-001|1.00e+000|1|0.3|STX1A;|</td></tr>
<tr><td>KEGG</td><td>hsa04140</td><td>Regulation of autophagy</td><td>9.07e-001|1.00e+000|2|0.5|IFNG;PRKAA1;|</td></tr>
<tr><td>KEGG</td><td>hsa04141</td><td>Protein processing in endoplasmic reticulum</td><td>9.73e-001|1.00e+000|10|0.6|BAK1;BAX;BCL2;EIF2AK1;ERO1B;HSP90AA1;HSP90AB1;P4HB;STUB1;VCP;
|</td></tr>
<tr><td>KEGG</td><td>hsa04142</td><td>Lysosome</td><td>9.94e-001|1.00e+000|5|0.4|AP3D1;CLN3;GALC;SLC11A1;SLC11A2;|</td></tr>
<tr><td>KEGG</td><td>hsa04144</td><td>Endocytosis</td><td>8.55e-001|1.00e+000|16|0.8|ADRBK1;ADRBK2;CAV3;CDC42;CXCR4;DNM3;FGFR2;FOLR1;FOLR2;NEDD4;
NEDD4L;PLD1;PLD2;PML;SMAD7;TGFB2;|</td></tr>
<tr><td>KEGG</td><td>hsa04145</td><td>Phagosome</td><td>1.00e+000|1.00e+000|2|0.1|CYBA;NOS1;|</td></tr>
<tr><td>KEGG</td><td>hsa04146</td><td>Peroxisome</td><td>8.78e-002|2.56e-001|12|1.5|ACOX1;ACSL1;AGXT;BAAT;CAT;DAO;HACL1;NOS2;NUDT12;PAOX;
PIPOX;XDH;|</td></tr>
</table>

KEGG	hsa04150	mTOR signaling pathway	9.42e-001|1.00e+000|3|0.5|HIF1A;IGF1;PRKAA1;|
KEGG	hsa04151	PI3K-Akt signaling pathway	9.78e-001|1.00e+000|24|0.7|ANGPT1;BAD;BCL2;BCL2L11;BRCA1;CDKN1A;CREB1;EPO;FASLG;FGF10;<br>FGF23;FGFR2;HSP90AA1;HSP90AB1;IGF1;IL4;INSR;NGFR;NOS3;PPP2CA;<br>PPP2CB;PRKAA1;PRLR;RXRA;|
KEGG	hsa04152	AMPK signaling pathway	4.41e-001|8.14e-001|13|1.1|CFTR;CREB1;FBP1;IGF1;INSR;LEP;PPARG;PPP2CA;PPP2CB;PRKAA1;<br>PRKAB1;PRKAB2;SIRT1;|
KEGG	hsa04210	Apoptosis	4.69e-001|8.59e-001|9|1.1|AIFM1;APAF1;BAD;BAX;BCL2;FADD;FAS;FASLG;RIPK1;|
KEGG	hsa04260	Cardiac muscle contraction	9.31e-006|7.83e-005|21|2.8|ATP1A1;CACNA1C;CACNA1D;CACNA2D1;CACNA2D2;COX1;COX2;COX3;COX4I1;COX5A;<br>COX5B;COX6A2;COX6B1;COX6C;COX7A1;COX7B;COX7C;COX8A;RYR2;SLC8A1;<br>TNNC1;|
KEGG	hsa04261	Adrenergic signaling in cardiomyocytes	8.93e-002|2.57e-001|20|1.3|ATP1A1;BCL2;CACNA1C;CACNA1D;CACNA2D1;CACNA2D2;CAMK2D;CAMK2G;CREB1;KCNQ1;<br>PLN;PPP2CA;PPP2CB;RYR2;SCN1B;SCN4B;SCN5A;SCN7A;SLC8A1;TNNC1;<br>|
KEGG	hsa04270	Vascular smooth muscle contraction	3.93e-001|7.69e-001|13|1.1|ADORA2A;ADORA2B;ARAF;CACNA1C;CACNA1D;GUCY1B3;KCNMA1;NPR1;NPR2;PLA2G1B;<br>PRKCD;ROCK1;ROCK2;|
KEGG	hsa04310	Wnt signaling pathway	9.34e-001|1.00e+000|9|0.6|CAMK2D;CAMK2G;CTNNB1;PPARD;ROCK2;WNT10B;WNT2;WNT2B;WNT4;|
KEGG	hsa04330	Notch signaling pathway	8.62e-001|1.00e+000|3|0.6|HDAC1;HDAC2;SNW1;|
KEGG	hsa04340	Hedgehog signaling pathway	2.28e-001|5.17e-001|7|1.4|BMP4;SHH;SMO;WNT10B;WNT2;WNT2B;WNT4;|
KEGG	hsa04350	TGF-beta signaling pathway	2.54e-001|5.59e-001|10|1.3|ACVR1;ACVR1B;AMHR2;BMP4;IFNG;PPP2CA;PPP2CB;ROCK1;SMAD7;TGFB2;<br>|
KEGG	hsa04360	Axon guidance	9.36e-001|1.00e+000|8|0.7|ABL1;CDC42;CXCR4;EPHA4;ROCK1;ROCK2;SEMA4D;SLIT2;|

<table>
<tr><td>KEGG</td><td>hsa04370</td><td>VEGF signaling pathway</td><td>7.26e-001|1.00e+000|5|0.8|BAD;CDC42;NOS3;PLCG2;PTGS2;|</td></tr>
<tr><td>KEGG</td><td>hsa04380</td><td>Osteoclast differentiation</td><td>8.97e-001|1.00e+000|9|0.7|CREB1;CYBA;IFNG;LILRB1;NFKB2;PLCG2;PPARG;SPI1;TGFB2;|</td></tr>
<tr><td>KEGG</td><td>hsa04390</td><td>Hippo signaling pathway</td><td>8.31e-001|1.00e+000|12|0.8|BMP4;CTNNB1;DLG4;PPP2CA;PPP2CB;SMAD7;SNAI2;TGFB2;WNT10B;WNT2;
WNT2B;WNT4;|</td></tr>
<tr><td>KEGG</td><td>hsa04510</td><td>Focal adhesion</td><td>9.99e-001|1.00e+000|9|0.5|ACTN3;BAD;BCL2;CAV3;CDC42;CTNNB1;IGF1;ROCK1;ROCK2;|</td></tr>
<tr><td>KEGG</td><td>hsa04512</td><td>ECM-receptor interaction</td><td>1.00e+000|1.00e+000|1|0.1|AGRN;|</td></tr>
<tr><td>KEGG</td><td>hsa04514</td><td>Cell adhesion molecules (CAMs)</td><td>9.96e-001|1.00e+000|6|0.4|CDH3;LRRC4B;NLGN1;NRXN1;NRXN2;NRXN3;|</td></tr>
<tr><td>KEGG</td><td>hsa04520</td><td>Adherens junction</td><td>7.33e-001|1.00e+000|6|0.8|ACTN3;CDC42;CTNNB1;INSR;SNAI1;SNAI2;|</td></tr>
<tr><td>KEGG</td><td>hsa04530</td><td>Tight junction</td><td>9.59e-001|1.00e+000|8|0.6|ACTN3;CDC42;CTNNB1;MAGI2;PPP2CA;PPP2CB;PRKCD;YBX3;|</td></tr>
<tr><td>KEGG</td><td>hsa04540</td><td>Gap junction</td><td>7.82e-001|1.00e+000|7|0.8|DRD1;DRD2;GUCY1B3;HTR2A;HTR2B;HTR2C;MAP2K5;|</td></tr>
<tr><td>KEGG</td><td>hsa04610</td><td>Complement and coagulation cascades</td><td>9.17e-001|1.00e+000|4|0.6|BDKRB2;PLAT;PLG;THBD;|</td></tr>
<tr><td>KEGG</td><td>hsa04611</td><td>Platelet activation</td><td>9.78e-001|1.00e+000|7|0.5|GUCY1B3;NOS3;PLCG2;PTGS1;ROCK1;ROCK2;STIM1;|</td></tr>
<tr><td>KEGG</td><td>hsa04612</td><td>Antigen processing and presentation</td><td>8.63e-001|1.00e+000|5|0.7|CD74;CREB1;HSP90AA1;HSP90AB1;IFNG;|</td></tr>
<tr><td>KEGG</td><td>hsa04620</td><td>Toll-like receptor signaling pathway</td><td>9.82e-001|1.00e+000|5|0.5|CCL5;FADD;RIPK1;TIRAP;TLR3;|</td></tr>
<tr><td>KEGG</td><td>hsa04621</td><td>NOD-like receptor signaling pathway</td><td>6.69e-001|1.00e+000|5|0.9|CCL2;CCL5;HSP90AA1;HSP90AB1;NOD2;|</td></tr>
</table>

<table>
<tr><td>KEGG</td><td>hsa04622</td><td>RIG-I-like receptor signaling pathway</td><td>9.94e-001|1.00e+000|2|0.3|FADD;RIPK1;|</td></tr>
<tr><td>KEGG</td><td>hsa04623</td><td>Cytosolic DNA-sensing pathway</td><td>9.89e-001|1.00e+000|2|0.3|CCL5;RIPK1;|</td></tr>
<tr><td>KEGG</td><td>hsa04630</td><td>Jak-STAT signaling pathway</td><td>9.89e-001|1.00e+000|8|0.5|EPO;IFNG;IL10;IL4;LEP;PIM1;PRLR;TPO;|</td></tr>
<tr><td>KEGG</td><td>hsa04640</td><td>Hematopoietic cell lineage</td><td>9.78e-001|1.00e+000|4|0.5|EPO;FCER2;IL4;TPO;|</td></tr>
<tr><td>KEGG</td><td>hsa04650</td><td>Natural killer cell mediated cytotoxicity</td><td>9.98e-001|1.00e+000|5|0.4|ARAF;FAS;FASLG;IFNG;PLCG2;|</td></tr>
<tr><td>KEGG</td><td>hsa04660</td><td>T cell receptor signaling pathway</td><td>9.93e-001|1.00e+000|4|0.4|CDC42;IFNG;IL10;IL4;|</td></tr>
<tr><td>KEGG</td><td>hsa04662</td><td>B cell receptor signaling pathway</td><td>9.99e-001|1.00e+000|1|0.1|PLCG2;|</td></tr>
<tr><td>KEGG</td><td>hsa04664</td><td>Fc epsilon RI signaling pathway</td><td>9.73e-001|1.00e+000|3|0.4|IL4;PLCG2;PRKCD;|</td></tr>
<tr><td>KEGG</td><td>hsa04666</td><td>Fc gamma R-mediated phagocytosis</td><td>9.52e-001|1.00e+000|5|0.6|CDC42;PLCG2;PLD1;PLD2;PRKCD;|</td></tr>
<tr><td>KEGG</td><td>hsa04668</td><td>TNF signaling pathway</td><td>3.91e-001|7.69e-001|12|1.1|CCL2;CCL5;CREB1;EDN1;FADD;FAS;LTA;MAGI2;MMP9;NOD2;
PTGS2;RIPK1;|</td></tr>
<tr><td>KEGG</td><td>hsa04670</td><td>Leukocyte transendothelial migration</td><td>8.30e-001|1.00e+000|9|0.8|ACTN3;CDC42;CTNNB1;CXCR4;CYBA;MMP9;PLCG2;ROCK1;ROCK2;|</td></tr>
<tr><td>KEGG</td><td>hsa04672</td><td>Intestinal immune network for IgA production</td><td>8.42e-001|1.00e+000|3|0.7|CXCR4;IL10;IL4;|</td></tr>
<tr><td>KEGG</td><td>hsa04710</td><td>Circadian rhythm</td><td>3.15e-001|6.56e-001|4|1.4|CREB1;PRKAA1;PRKAB1;PRKAB2;|</td></tr>
<tr><td>KEGG</td><td>hsa04713</td><td>Circadian entrainment</td><td>8.31e-002|2.45e-001|14|1.5|CACNA1C;CACNA1D;CAMK2D;CAMK2G;CREB1;GRIN1;GRIN2A;GRIN2B;GRIN2C;GRIN2D;
GUCY1B3;NOS1;RYR2;RYR3;|</td></tr>
</table>

KEGG	hsa04720	Long-term potentiation	2.05e-001|4.90e-001|9|1.4|ARAF;CACNA1C;CAMK2D;CAMK2G;GRIN1;GRIN2A;GRIN2B;GRIN2C;GRIN2D;|
KEGG	hsa04721	Synaptic vesicle cycle	4.22e-001|7.92e-001|7|1.1|CACNA1A;CACNA1B;DNM3;RAB3A;SLC17A7;SLC32A1;STX1A;|
KEGG	hsa04722	Neurotrophin signaling pathway	5.15e-001|8.86e-001|12|1.0|ABL1;BAD;BAX;BCL2;CAMK2D;CAMK2G;CDC42;FASLG;MAP2K5;NGFR;<br>PLCG2;PRKCD;|
KEGG	hsa04723	Retrograde endocannabinoid signaling	1.07e-005|8.67e-005|25|2.5|CACNA1A;CACNA1B;CACNA1C;CACNA1D;CNR1;GABRA1;GABRA2;GABRA3;GABRA4;GABRA5;<br>GABRA6;GABRB1;GABRB2;GABRB3;GABRD;GABRE;GABRG1;GABRG2;GABRG3;GABRP;<br>GABRQ;NAPEPLD;PTGS2;SLC17A7;SLC32A1;|
KEGG	hsa04724	Glutamatergic synapse	1.52e-003|7.84e-003|22|2.0|ADRBK1;ADRBK2;CACNA1A;CACNA1C;CACNA1D;DLG4;GLUL;GRIN1;GRIN2A;GRIN2B;<br>GRIN2C;GRIN2D;GRIN3A;GRIN3B;PLD1;PLD2;SHANK3;SLC17A7;SLC1A1;SLC1A3;<br>SLC1A6;SLC38A3;|
KEGG	hsa04725	Cholinergic synapse	2.04e-001|4.90e-001|14|1.3|ACHE;BCL2;CACNA1A;CACNA1B;CACNA1C;CACNA1D;CAMK2D;CAMK2G;CHRNA3;CHRNB2;<br>CHRNB4;CREB1;KCNQ1;SLC5A7;|
KEGG	hsa04726	Serotonergic synapse	6.88e-005|4.88e-004|25|2.3|ALOX5;ARAF;CACNA1A;CACNA1B;CACNA1C;CACNA1D;DDC;GABRB1;GABRB2;GABRB3;<br>HTR1A;HTR1B;HTR1D;HTR2A;HTR2B;HTR2C;HTR7;KCND2;MAOA;MAOB;<br>PTGS1;PTGS2;SLC6A4;TPH1;TPH2;|
KEGG	hsa04727	GABAergic synapse	7.20e-007|7.78e-006|25|2.9|ABAT;CACNA1A;CACNA1B;CACNA1C;CACNA1D;GABRA1;GABRA2;GABRA3;GABRA4;GABRA5;<br>GABRA6;GABRB1;GABRB2;GABRB3;GABRD;GABRE;GABRG1;GABRG2;GABRG3;GABRP;<br>GABRQ;GLUL;HAP1;SLC32A1;SLC38A3;|
KEGG	hsa04728	Dopaminergic synapse	1.58e-003|7.97e-003|24|1.9|CACNA1A;CACNA1B;CACNA1C;CACNA1D;CALY;CAMK2D;CAMK2G;COMT;CREB1;DDC;<br>DRD1;DRD2;DRD3;DRD4;DRD5;GRIN2A;GRIN2B;MAOA;MAOB;PPP2CA;<br>PPP2CB;SCN1A;SLC6A3;TH;|
KEGG	hsa04730	Long-term depression	2.30e-001|5.17e-001|8|1.4|ARAF;CACNA1A;CRH;GUCY1B3;IGF1;NOS1;PPP2CA;PPP2CB;|
KEGG	hsa04740	Olfactory transduction	1.00e+000|1.00e+000|3|0.1|ADRBK2;CAMK2D;CAMK2G;|

<table>
<tr><td>KEGG</td><td>hsa04742</td><td>Taste transduction</td><td>8.89e-001|1.00e+000|3|0.6|CACNA1A;CACNA1B;KCNB1;|</td></tr>
<tr><td>KEGG</td><td>hsa04750</td><td>Inflammatory mediator regulation of TRP channels</td><td>3.00e-002|1.13e-001|16|1.6|BDKRB2;CAMK2D;CAMK2G;F2RL1;HTR2A;HTR2B;HTR2C;IGF1;PLCG2;PRKCD;
PTGER2;PTGER4;TRPA1;TRPM8;TRPV1;TRPV3;|</td></tr>
<tr><td>KEGG</td><td>hsa04810</td><td>Regulation of actin cytoskeleton</td><td>9.98e-001|1.00e+000|10|0.5|ACTN3;ARAF;BDKRB2;CDC42;FGF10;FGF23;FGFR2;NCKAP1L;ROCK1;ROCK2;
|</td></tr>
<tr><td>KEGG</td><td>hsa04910</td><td>Insulin signaling pathway</td><td>8.96e-001|1.00e+000|10|0.7|ARAF;BAD;FBP1;INSR;PDE3A;PDE3B;PRKAA1;PRKAB1;PRKAB2;PYGL;
|</td></tr>
<tr><td>KEGG</td><td>hsa04911</td><td>Insulin secretion</td><td>2.17e-001|5.08e-001|11|1.3|ABCC8;ATP1A1;CACNA1C;CACNA1D;CAMK2D;CAMK2G;CREB1;KCNMA1;RAB3A;RYR2;
STX1A;|</td></tr>
<tr><td>KEGG</td><td>hsa04912</td><td>GnRH signaling pathway</td><td>5.53e-001|9.44e-001|9|1.0|CACNA1C;CACNA1D;CAMK2D;CAMK2G;CDC42;GNRH1;PLD1;PLD2;PRKCD;|</td></tr>
<tr><td>KEGG</td><td>hsa04913</td><td>Ovarian Steroidogenesis</td><td>9.06e-003|3.96e-002|11|2.2|AKR1C3;ALOX5;CYP11A1;CYP19A1;CYP1A1;HSD17B1;HSD17B2;IGF1;INSR;PTGS2;
STAR;|</td></tr>
<tr><td>KEGG</td><td>hsa04914</td><td>Progesterone-mediated oocyte maturation</td><td>7.51e-001|1.00e+000|7|0.8|ARAF;HSP90AA1;HSP90AB1;IGF1;PDE3A;PDE3B;PGR;|</td></tr>
<tr><td>KEGG</td><td>hsa04915</td><td>Estrogen signaling pathway</td><td>8.71e-001|1.00e+000|7|0.7|CREB1;ESR1;HSP90AA1;HSP90AB1;MMP9;NOS3;PRKCD;|</td></tr>
<tr><td>KEGG</td><td>hsa04916</td><td>Melanogenesis</td><td>2.83e-001|6.06e-001|12|1.2|CAMK2D;CAMK2G;CREB1;CTNNB1;DCT;EDN1;TYR;TYRP1;WNT10B;WNT2;
WNT2B;WNT4;|</td></tr>
<tr><td>KEGG</td><td>hsa04917</td><td>Prolactin signaling pathway</td><td>9.77e-001|1.00e+000|3|0.4|ESR1;PRLR;TH;|</td></tr>
<tr><td>KEGG</td><td>hsa04918</td><td>Thyroid hormone synthesis</td><td>7.20e-001|1.00e+000|6|0.8|ATP1A1;CREB1;GPX7;GSR;IYD;TPO;|</td></tr>
</table>

KEGG	hsa04919	Thyroid hormone signaling pathway	2.73e-001|5.90e-001|14|1.2|ATP1A1;BAD;BMP4;CTNNB1;ESR1;HDAC1;HDAC2;HIF1A;MED1;NCOA1;<br>PLCG2;PLN;RXRA;WNT4;|
KEGG	hsa04920	Adipocytokine signaling pathway	6.94e-001|1.00e+000|6|0.9|ACSL1;LEP;PRKAA1;PRKAB1;PRKAB2;RXRA;|
KEGG	hsa04921	Oxytocin signaling pathway	5.94e-002|1.94e-001|22|1.4|CACNA1C;CACNA1D;CACNA2D1;CACNA2D2;CAMK2D;CAMK2G;CDKN1A;GUCY1B3;MAP2K5;NOS3;<br>NPR1;NPR2;OXT;OXTR;PRKAA1;PRKAB1;PRKAB2;PTGS2;ROCK1;ROCK2;<br>RYR2;RYR3;|
KEGG	hsa04960	Aldosterone-regulated sodium reabsorption	3.34e-001|6.89e-001|5|1.3|ATP1A1;IGF1;INSR;NEDD4L;NR3C2;|
KEGG	hsa04961	Endocrine and other factor-regulated calcium reabsorption	9.22e-002|2.58e-001|8|1.7|ATP1A1;BDKRB2;CALB1;DNM3;ESR1;KL;SLC8A1;VDR;|
KEGG	hsa04962	Vasopressin-regulated water reabsorption	9.90e-001|1.00e+000|1|0.2|CREB1;|
KEGG	hsa04964	Proximal tubule bicarbonate reclamation	1.83e-001|4.52e-001|4|1.7|AQP1;ATP1A1;SLC25A10;SLC38A3;|
KEGG	hsa04970	Salivary secretion	8.81e-001|1.00e+000|6|0.7|ATP1A1;GUCY1B3;KCNMA1;LYZ;NOS1;RYR3;|
KEGG	hsa04971	Gastric acid secretion	7.57e-001|1.00e+000|6|0.8|ATP1A1;CAMK2D;CAMK2G;CFTR;KCNQ1;SST;|
KEGG	hsa04972	Pancreatic secretion	8.19e-001|1.00e+000|7|0.8|ATP1A1;CFTR;KCNMA1;KCNQ1;PLA2G1B;PNLIPRP2;RYR2;|
KEGG	hsa04973	Carbohydrate digestion and absorption	9.44e-001|1.00e+000|2|0.5|ATP1A1;CACNA1D;|
KEGG	hsa04974	Protein digestion and absorption	4.98e-001|8.79e-001|9|1.1|ATP1A1;KCNQ1;SLC1A1;SLC1A5;SLC36A1;SLC3A1;SLC7A7;SLC7A8;SLC8A1;|
KEGG	hsa04975	Fat digestion and absorption	2.81e-002|1.08e-001|8|2.2|ABCA1;APOA1;DGAT2;FABP2;GOT2;MTTP;PLA2G1B;PNLIPRP2;|

KEGG	hsa04976	Bile secretion	9.02e-003|3.96e-002|14|2.0|ABCB1;ABCB11;ABCC2;AQP1;AQP8;ATP1A1;BAAT;CFTR;CYP3A4;NR1H4;<br>RXRA;SLCO1B1;SLCO1B3;SULT2A1;|
KEGG	hsa04977	Vitamin digestion and absorption	1.83e-001|4.52e-001|4|1.7|APOA1;CUBN;MMACHC;SLC5A6;|
KEGG	hsa04978	Mineral absorption	2.28e-001|5.17e-001|7|1.4|ATP1A1;HMOX1;S100G;SLC11A1;SLC11A2;SLC8A1;VDR;|
